# UFSRAT: Ultra-Fast Shape Recognition with Atom Types –The Discovery of Novel Bioactive Small Molecular Scaffolds for FKBP12 and 11βHSD1

**DOI:** 10.1371/journal.pone.0116570

**Published:** 2015-02-06

**Authors:** Steven Shave, Elizabeth A. Blackburn, Jillian Adie, Douglas R. Houston, Manfred Auer, Scott P. Webster, Paul Taylor, Malcolm D. Walkinshaw

**Affiliations:** 1 Centre for Translational and Chemical Biology, University of Edinburgh, Edinburgh, United Kingdom; 2 University/BHF Centre for Cardiovascular Science, The Queen’s Medical Research Institute, University of Edinburgh, Edinburgh, United Kingdom; National Institute for Medical Research, Medical Research Council, London, UNITED KINGDOM

## Abstract

**Motivation:**

Using molecular similarity to discover bioactive small molecules with novel chemical scaffolds can be computationally demanding. We describe Ultra-fast Shape Recognition with Atom Types (UFSRAT), an efficient algorithm that considers both the 3D distribution (shape) and electrostatics of atoms to score and retrieve molecules capable of making similar interactions to those of the supplied query.

**Results:**

Computational optimization and pre-calculation of molecular descriptors enables a query molecule to be run against a database containing 3.8 million molecules and results returned in under 10 seconds on modest hardware. UFSRAT has been used in pipelines to identify bioactive molecules for two clinically relevant drug targets; FK506-Binding Protein 12 and 11β-hydroxysteroid dehydrogenase type 1. In the case of FK506-Binding Protein 12, UFSRAT was used as the first step in a structure-based virtual screening pipeline, yielding many actives, of which the most active shows a K_D, app_ of 281 µM and contains a substructure present in the query compound. Success was also achieved running solely the UFSRAT technique to identify new actives for 11β-hydroxysteroid dehydrogenase type 1, for which the most active displays an IC50 of 67 nM in a cell based assay and contains a substructure radically different to the query. This demonstrates the valuable ability of the UFSRAT algorithm to perform scaffold hops.

**Availability and Implementation:**

A web-based implementation of the algorithm is freely available at http://opus.bch.ed.ac.uk/ufsrat/.

## Introduction

The concept of molecular similarity has been exploited in nearly all chemical fields and has been used to great effect in the pharmaceutical industry to reduce the massive cost of drug development [1–3]. When molecular similarity is employed in ligand-based virtual screening it offers the ability to carry out searches for actives where little is known about the drug receptor, only molecules which bind to it [4–8]. Structurally similar molecules can exhibit similar biological properties and may therefore bind to receptors, making the same or equivalent interactions as the native ligand [6, 9]. Molecular similarity and more specifically, scaffold hopping also provides a route to ‘rescue’ problematic drug leads which may well be inhibitors of a protein, but are unsuitable for further development due to problems with pharmacology, pharmacokinetics or patent issues [3, 10]. Scaffold hopping describes the discovery of a compound with the same or similar bioactivity as the query compound but with a different core molecular structure. Successful scaffold hopping methodologies commonly describe the virtual compound in a way that encodes both the 3D shape of the molecule and the electrostatic and hydrophobic properties. This is key to successful lead discovery because electrostatic and van der Waals interactions are very sensitive to bond geometry and distance. There is of course a direct correlation between the levels of detail encoded in molecular descriptors or force-field based approaches and computational resources. It is essential to develop algorithms that can succinctly capture the essential molecular features and then search very large databases in a computationally efficient manner. We have developed the idea of capturing molecular shape using parameters determined from the interatomic distance distributions first proposed by Ballester and Richards [11, 12] and incorporate these pre-calculated molecular descriptors into a searchable database of available compounds [13].

In this paper we describe the use of our UFSRAT algorithm (an expansion of the validated [14–19] USR technique) in virtual screening pipelines to identify inhibitors of two unrelated enzymes; FK506-Binding Protein 12 (FKBP12) and 11β-hydroxysteroid dehydrogenase type 1 (11β-HSD1). FKBP12 is a peptidyl-prolyl isomerase, catalysing protein folding [20–22] and is a therapeutic target for Parkinson’s and Alzheimer’s disease [23]. The enzyme 11β-HSD1 catalyses the intracellular biosynthesis of the active glucocorticoid steroid hormone cortisol which plays a central role in glucose homeostasis and the inflammatory response [24, 25]. Inhibitors of 11β-HSD1 have been investigated for targeting cardiometabolic diseases such as type-2 diabetes, as well as glaucoma, osteoporosis and Alzheimer’s disease. Cellular and direct binding assays show that UFSRAT successfully identified highly active non-steroid inhibitors with nanomolar activity.

## Methods

### Computational Methods: Ultra fast shape recognition with atom types

Applying the Ultra Fast Shape Recognition with Atom Types (UFSRAT) technique to a query molecule and a candidate molecule returns a measure of similarity between the two. This process consists of three steps: first shape and atom property descriptors are calculated for each molecule; second, the descriptors are compared using a scoring function and finally similar molecules are ranked by score. Ballester and Richards outlined Ultrafast Shape Recognition (USR) [11, 12] an algorithm that can be used to assess the shape similarity between two molecules. With this approach, no distinction is made between different types of atom. UFSRAT has been developed using USR as the base concept but additionally encodes hydrophobic and electrostatic information; features of the molecule that are important in molecular recognition.

**Calculating descriptors**. UFSRAT calculates 4 descriptor sets for each virtual molecule; each set encodes different features of the molecule. The distributions used to generate UFSRAT descriptors are: All atoms (shape), hydrophobic atoms, hydrogen bond acceptor atoms and hydrogen bond donor atoms.

**Figure 1:**
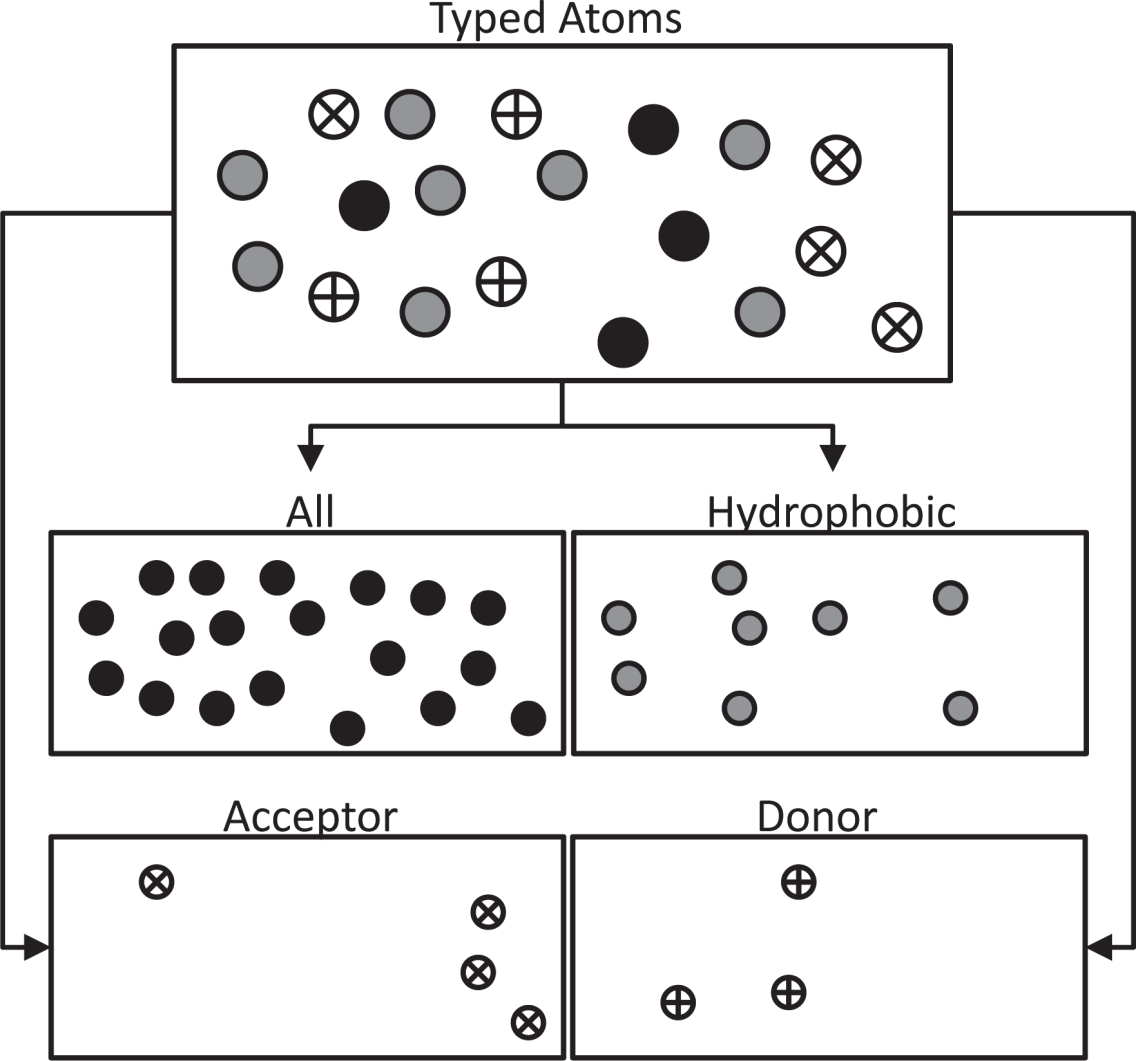
The four UFSRAT distributions from typed atoms. A molecule is broken down into four distributions, consisting of all atoms, hydrophobic, hydrogen bond acceptor and hydrogen bond donor.

**Figure 2:**
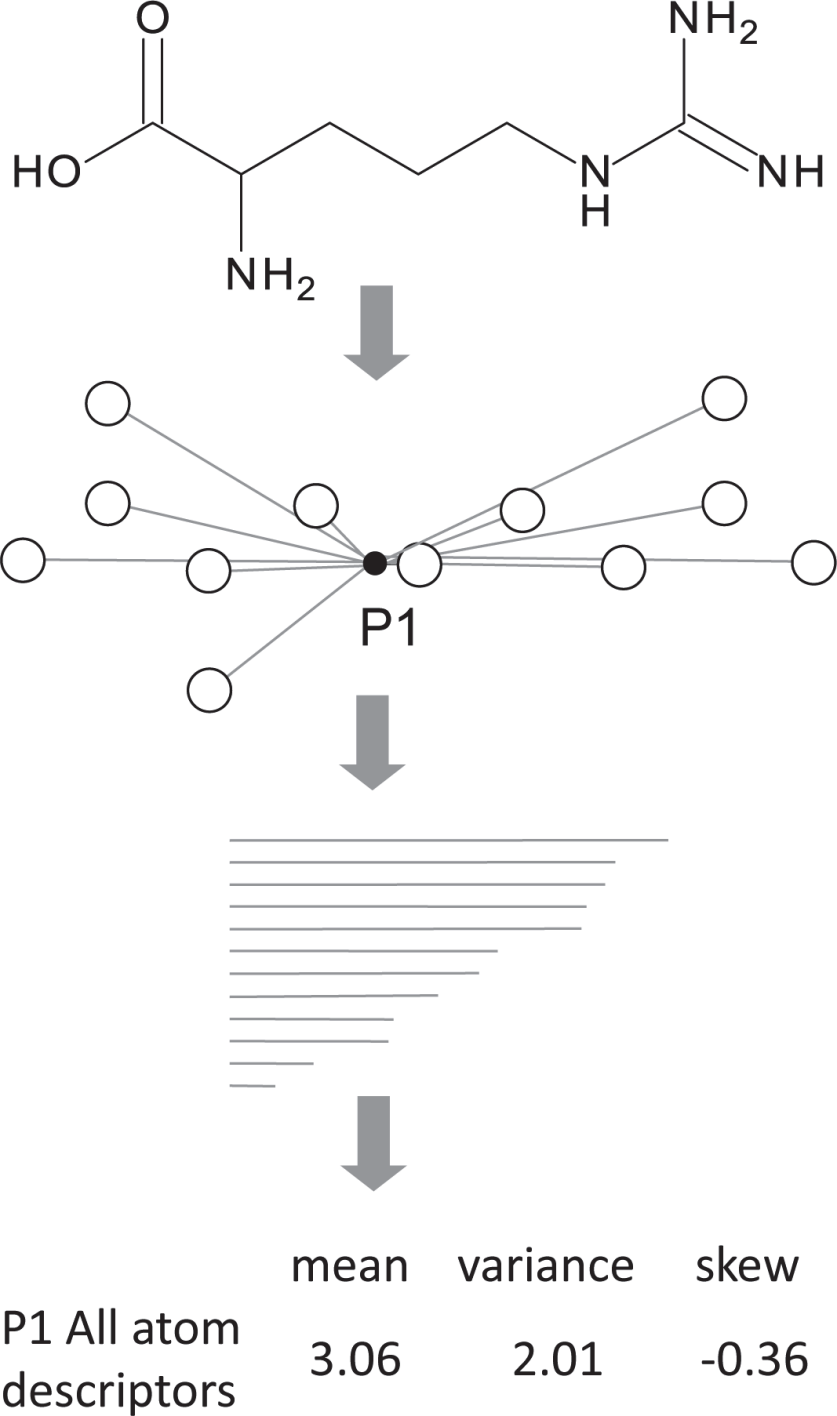
Generation of UFSRAT all atom descriptors for the P1 distribution. The atoms in a molecule are all considered as an array of points (black open circles). P1 represents the geometric centre of the atoms (black dot). Euclidean distances are calculated to all atoms from P1 (grey lines). The 3 descriptors for the P1 distribution are the mean, variance and skew of the distance distribution.

Determining which atoms within a molecule should be considered for each distribution requires atom type information to be calculated. Atom typing can be described as applying a user definable atomic mask. This is achieved using the same implementation present in the high throughput virtual screening code LIDAEUS [26, 27]. Fig. 1 illustrates the generation of UFSRAT distributions whilst Fig. 2 shows how the descriptors are calculated. 12 descriptors are calculated for each of the 4 distributions resulting in 48 descriptors for each molecule. This is an additional 36 compared to USR which only uses an all atom distribution. The 12 descriptors for each set are calculated from the geometric atom distributions. Fig. 2 illustrates a simplified all atom distribution descriptor generation process applied to a 2D small molecule (note that hydrogens are ignored). Calculating the 12 values takes the following form; (i) Atomic coordinates are used to define the centre of the volume occupied by the molecule as point 1 (P1). (ii) A distribution of Euclidean distances of all atoms to P1 is generated. (iii) From this distribution, 3 values are determined; the mean, variance and skew. These three values for all atoms relative to P1 make up the first 3 of the 12 descriptors. (iv) The closest atom to the geometric centre is defined as point 2 (P2). Similarly, the Euclidian distances of all atoms to P2 are calculated and the mean, variance and skew of this distribution are determined. These make up descriptor values 4, 5 and 6. (v) The furthest atom from P2 is defined as point 3 (P3). Again, the mean, variance and skew of the distribution made up of Euclidian distances of all atoms to P3 go to make up values 7, 8 and 9 of the descriptors. (vi) Finally, point 4 (P4) is the furthest atom from P3. To make up the final three values of the twelve descriptors, the Euclidian distance of all atoms from this point are calculated and then the mean, variance and skew calculated from this distribution.

**Figure 3:**
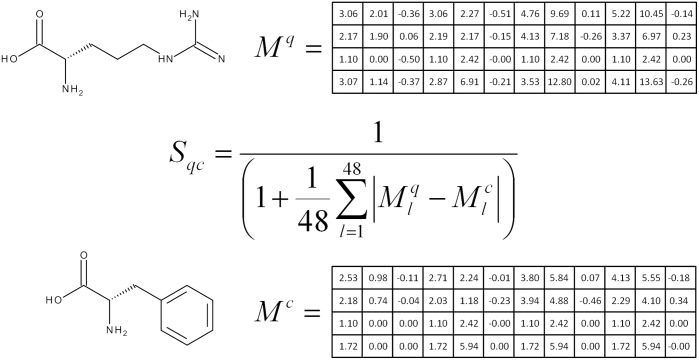
The UFSRAT Scoring function. UFSRAT descriptor values have been calculated for the molecules shown and their values input to the scoring function shown. Sqc is the similarity score between the two molecules q and c (query and candidate), M being a vector representing the 48 geometric distribution descriptors.

**Comparing molecular descriptors**. The 48 geometric distribution descriptors are pre-calculated for each candidate molecule in the database and stored as vectors M^c^. M^q^ represents the vector for the query molecule. The USFRAT scoring function is used to generate a similarity score, S_qc_, between the query molecule and each candidate molecule in the database. This is illustrated in Fig. 3. The similarity score S_qc_ is a single numerical metric pertaining to the predicted similarity of the molecules. As candidate molecules are tested against the query, a sorted list of the top matches is kept and returned as the result upon completion.

**Ranking compounds by similarity**. The similarity scoring function S_qc_ is an extension of the method used by USR, calculating a weighted difference for each distribution and scaling the result between 0 and 1. UFSRAT is therefore typically used to screen a large database of potential candidate molecules against a query resulting in a ranked list of arbitrary length such as the top 500 similars.

**UFSRAT—execution**. A high speed version UFSRAT is currently deployed on the University of Edinburgh’s EDULISS system [13]. EDULISS is a web based molecular database mining tool. Containing 3.8 million unique compounds, the system employs a number of methods for similarity searching. The inclusion of UFSRAT into this system as a similarity method has enabled the pre calculation of UFSRAT descriptors for each of the 3.8 million molecules. Storage of pre-calculated descriptors is achieved using one binary data file. Storing all required information along with a unique identifier for a molecule requires 200 bytes. This is made up of 8 bytes for the identifier, and 192 bytes for the 48 descriptor values. UFSRAT therefore requires 730 megabytes of data to represent 3.8 million compounds in a condensed form suitable for similarity searching. Pre-calculating the descriptors in this way enables UFSRAT to read in the query molecule supplied by the user and then only ever calculate descriptors for the query. The scoring is then performed on the pre-calculated descriptors along with the newly generated query molecule descriptors. Upon completion, unique identifiers are used to retrieve top scoring molecules from the database and return them to the user in the form of a multi-molecule MDL-SD file. This approach results in a run time of around 8 seconds to query a molecule against the whole database of candidate molecules and supply results to the user using a modest 2010 linux-based server running on 1 processor. Linear multiprocessor speedup has also been achieved by removing disk operations from timings and parallelization using the OpenMP shared memory paradigm [28] on up to 8 processors.

### The FKBP12 virtual screening pipeline

**Figure 4:**
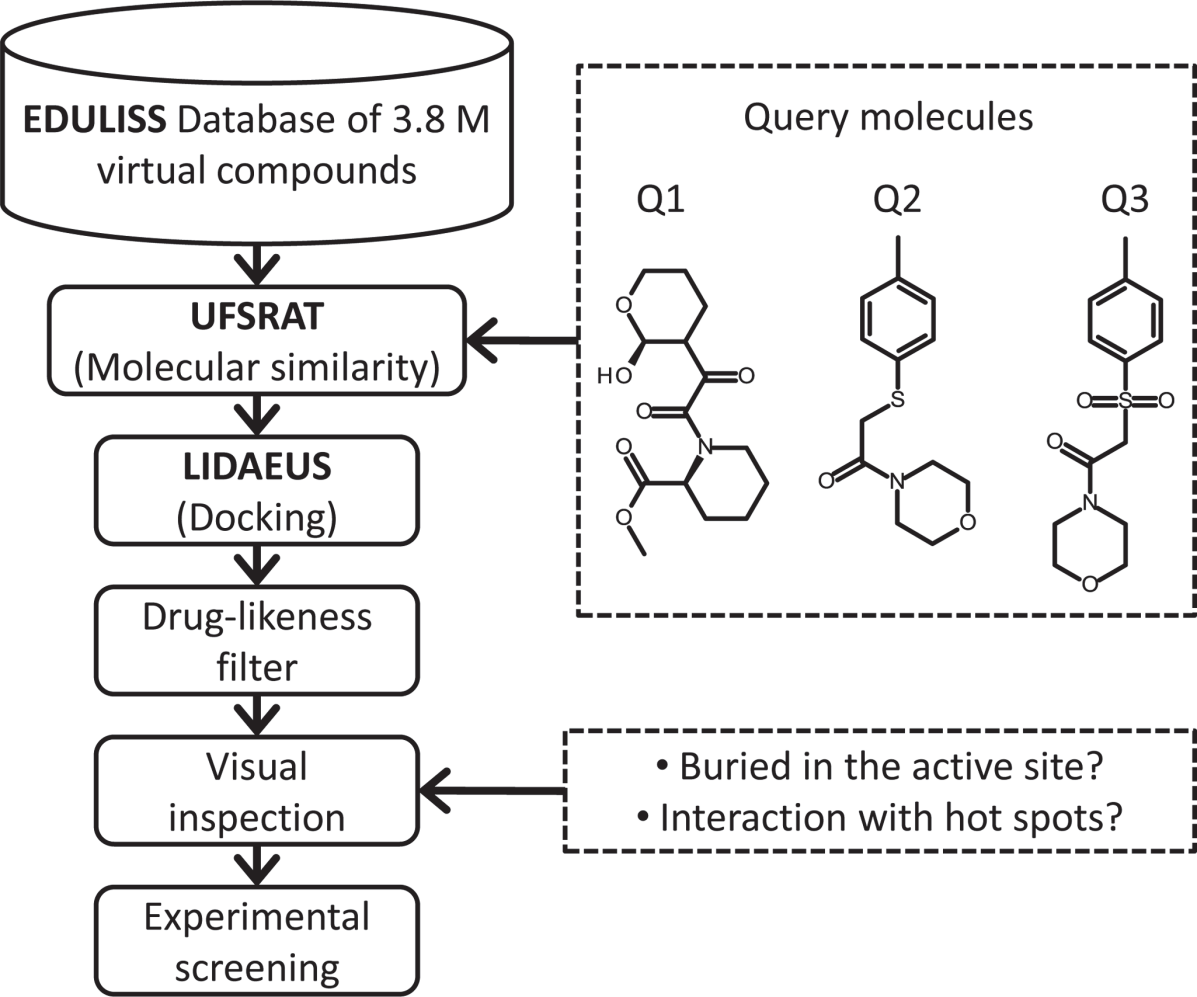
The UFSRAT workflow.

UFSRAT was used to select compounds similar to known inhibitors of FKBP12 from the EDULISS database of small molecules. Three well characterised FKBP12 inhibitors were used to generate UFSRAT queries. The results of running these queries with UFSRAT acted as the starting point in a structure-based virtual screening pipeline (See Fig. 4).

Around 50% of atoms contained within the high affinity macrocyclic immunosuppressants rapamycin and FK506 bind to FKBP12; the remainder exert a pharmacological effect by interacting with a second protein; mTor and calcineurin respectively [29]. Both rapamycin and FK506 share the same FKBP12 binding motif that occupies the majority of the active site making four hydrogen bonds. A high resolution crystal structure of FKBP12 in complex with rapamycin (2DG3.pdb), was used to select the buried fragment of rapamycin made up of atoms within 3.5 Å of FKBP12 [30], defining the query molecule Q1 (See Fig. 4). Two additional queries were selected from compounds from an NMR screen: 2-[(4-methylphenyl)sulfanyl]-1-(morpholin-4-yl)ethan-1-one, Q2, and (2-[(4-methylbenzene)-sulfonyl]-1-(morpholin-4-yl)ethan-1-one), Q3, (Fig. 4) [31]. The top 500 hits from UFSRAT were saved from each query (1500 total, 0.04% of the database) and docked into template 2DG3.pdb using the program LIDAEUS: grid spacing 0.6 Å; docking resolution parameter 0.04 Å [26, 32]. Ligands matching the shape and atom types of the site-points were ranked according to scores describing the enthalpy and buriedness of the interaction and then filtered for drug-likeness using Lipinski’s criteria and descriptors pre-calculated in the EDULISS data-base [33]. Manual inspection using PyMol identified ligands with predicted poses occupying the base of the pocket and made at least one hydrogen bond with Tyr82 or Ile56; hot spot residues in the active site of FKBP12. Hits containing the same scaffold as the query were not selected for purchase.

### The 11β-HSD1 virtual screening pipeline

**Figure 5:**
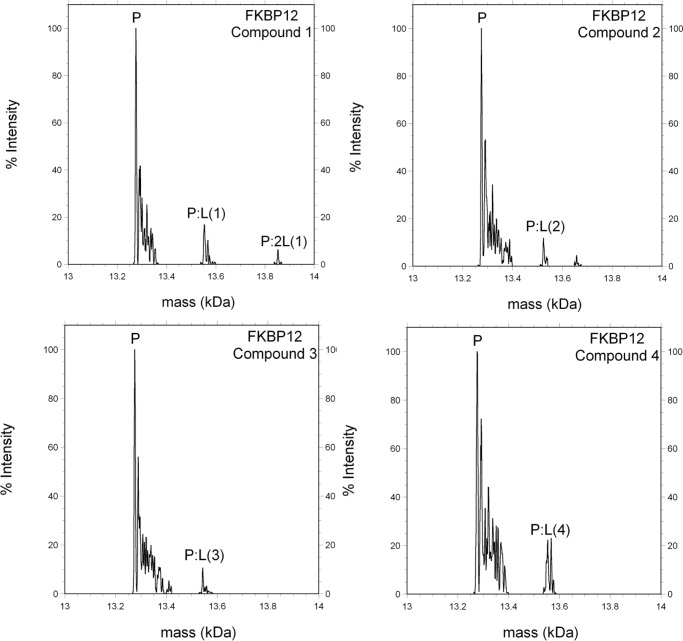
Carbenoxolone, 20nM inhibitor of 11β-HSD1.

Carbenoxolone, a potent bi-directional inhibitor of 11β-HSD1, was used to generate a UFSRAT query [34] (see Fig. 5). The structure of carbenoxolone in complex with the enzyme is available in the protein data bank with entry ID 2BEL. Candidate molecules were selected from the EDULISS database, the FKBP12 workflow outlined in Fig. 4 was followed with the exception of the docking step.

### Testing virtual hits

All reagents were of the highest grade available. Compounds were purchased from: Chembridge Corp., USA; InterBioScreen, Russia; Sigma Aldrich, UK; Specs, The Netherlands and TimTec, USA. Recombinant human FKBP12 was expressed and purified as described in [35]. The purification of recombinant human 11β-HSD1 is described by Adie [36]. Visualization of 3D structures was performed using PyMOL [37] (open source, DeLano Scientific LLC).

**Thermal denaturation fluorescence (TDF)**. A fluorescence based thermal denaturation assay was used to detect binding to FKBP12 essentially as described in the study of Lo [38]. Final conditions were: 50 mM ammonium acetate, pH 7.0; 10 μM FKBP12; 250 μM screening compound and 5x SYPRO orange dye (Invitrogen). The compound used for Q2, 2-[(4-methylphenyl)sulfanyl]-1-(morpholin-4-yl)ethan-1-one, acted as a positive control (K_d_ = 15 μM, 27°C) [31]. Protein was melted over the temperature range 20 to 80°C; 0.5°C increment, 30s hold. Compounds were screened for fluorescence in the absence of protein; all samples were solvent matched and repeated in triplicate on 3 separate experiments. Apparent equilibrium dissociation constants (K_d_^app^) for the interaction between the protein and ligands were calculated from the change in mid-point melting temperature of the protein (T_m_) on addition of ligand and transformed to 25°C using a benchmark value for the enthalpy of ligand binding of -5 kcal∙mol^-1^ [39].

**Electrospray ionization mass spectrometry**. Electrospray-ionization mass spectrometry (ESI-MS) was used to detect binding to FKBP12. Data was collected on a LCQ^TM^DECA ion-trap instrument (ThermoQuest) run in the positive ion mode. The analyte was introduced by direct infusion and the following operating parameters were employed: nitrogen flow 100units, analyte flow 6 μl∙min^-1^, capillary voltage 3.5 kV, cone voltage 25 V and capillary temperature 50°C. Spectrum were averaged over 300 scans (2s/ scan) and processed using the Bioworks Browser V 3.0 software package and ProMass 2.5 (ThermoScientific). The analyte solution consisted of 100 μM ligand, 5 μM FKBP12 in 10% methanol (v/v), 10 mM ammonium acetate, pH 6.8 incubated for 10 minutes at room temperature. K_d_^app^ for the interaction between the protein and ligand were calculated from the ratio of the intensity of the ion signal from free protein and protein in complex with ligand using the method of Tjernberg [40].

**Testing for inhibition of 11β-HSD1 reductase activity in cells**. Candidate compounds were tested for inhibition of the conversion of cortisone to cortisol by 11β-HSD1 expressed in HEK-293 cells stably transfected with the full length human hsd11b1 gene as previously described [41]. An established scintillation proximity assay (SPA) was carried out in a 96-well plate format, with appropriate controls [42]. Experiments were set up in triplicate with 8 inhibitor concentration points per compound, spanning 3.5 orders of magnitude. Final solution conditions: 40 nM [^3^H]-cortisone; 50 mM Tris pH 8.0, 50 mM NaCl; 1% DMSO. The anti-cortisol antibody was purchased from HyTest, Finland; radiometric quantification was carried out on a Perkin Elmer TopCount instrument. The percentage inhibition was determined relative to a non-inhibited control and the IC50 determined from a plot of percentage inhibition against compound concentration. Data were fitted global nonlinear regression using Kaleidagraph V4.0 (Synergy Software).

**Testing for inhibition of 11β-HSD1 dehydrogenase activity**. Candidate compounds were tested for inhibition of the conversion of cortisol to cortisone by recombinant h11β-HSD1. The dehydrogenation of cortisol is accompanied by the reduction of NADP^+^ to NADPH; this was followed spectrophotometrically and scaled to a NADPH calibration curve. The excitation and emission wavelengths were 340 and 458 nm. (SpectraMax M5, Molecular Devices). The experimental conditions were 500 μM NADP, 1 μM 11β-HSD1, 200 μM cortisol 50 mM Tris, pH 7.7, 50 mM NaCl; 25°C. Reactions were carried out in sextuplicate. K_i_^app^ were derived from a fit of the rate of the reaction as a function of inhibitor concentration; Kaleidagraph, V4.0 [43]

**Performance comparison of UFSRAT with USR using the DUD-E dataset**. The Directory of Useful Decoys: Extended (DUD-E) dataset [44] was used to compare the enrichment performance of UFSRAT with USR. We also included the popular topological fingerprint ECFP4 [45] as implemented in the RDKit toolkit [46]. Using 102 crystallographic ligands as query molecules for targets within the DUD-E dataset, collections of molecules containing known actives for each target along with random small molecules were run using each similarity method. Statistics were then gathered on recall of known actives within the top scoring 0.5%, 1%, 2%, and 5% of results. An enrichment score was then given to each method against each target. An enrichment score of 1 denoting the number of hits found would be expected by random picking of compounds. A score of 2 denoting double the expected recall over random picking and so on.

## Results

### Inhibitors of FKBP12

Of the 10 compounds tested, 4 compounds were hits in both the thermal denaturation fluorescence and ESI-MS screening assays. A compound was defined as a hit if it raised the mid-point melting temperature of the protein by at least 0.5°C and in ESI-MS if hits were seen to fly as an ion consistent with the mass of the protein-ligand complex. Structures of compounds **1** to **4** are available in S3 Fig. and affinity data is shown in Table 1. ESI-MS spectra of FKBP12 ligand complex are shown in Fig. 6.

**Table 1:** Binding data for FKBP12.

**Compound ID**	**IUPAC name**	TDF* K_d_^app^ (μM), T_m_*(transformed 25°C)*	ESI-MS* K_d_^app^ (μM), 50°C
**1**	2-(phenacylcarbamoyl)benzoic acid	548±38 *(252)*	313±30
**2**	N-(2-hydroxy-1-methyl-2-phenyl-ethyl)benzamide	575±70 *(266)*	399±45
**3**	3-chloro-N-(2-hydroxy-2-phenyl-ethyl)benzamide	568±84 *(263)*	383±52
**4**	N-(2-acetamidophenyl)-3-phenyl-propanamide	648±88*(303)*	281±42
**Q2**	1-morpholino-2-(p-tolylsulfanyl)ethanone	28±8 *(6)^#^*	197±74

Molecules 1, 2 and 3 were retrieved using query molecules Q1, Q2 and Q3 and the UFSRAT technique, screened against the EDULISS database. Binding of the top hits was tested using thermal denaturation (TDI) and mass spectrometry (ESI-MS).

* Recombinant human FKBP12

# K_d_ 15 μM, 27°C [31]

**Figure 6:**
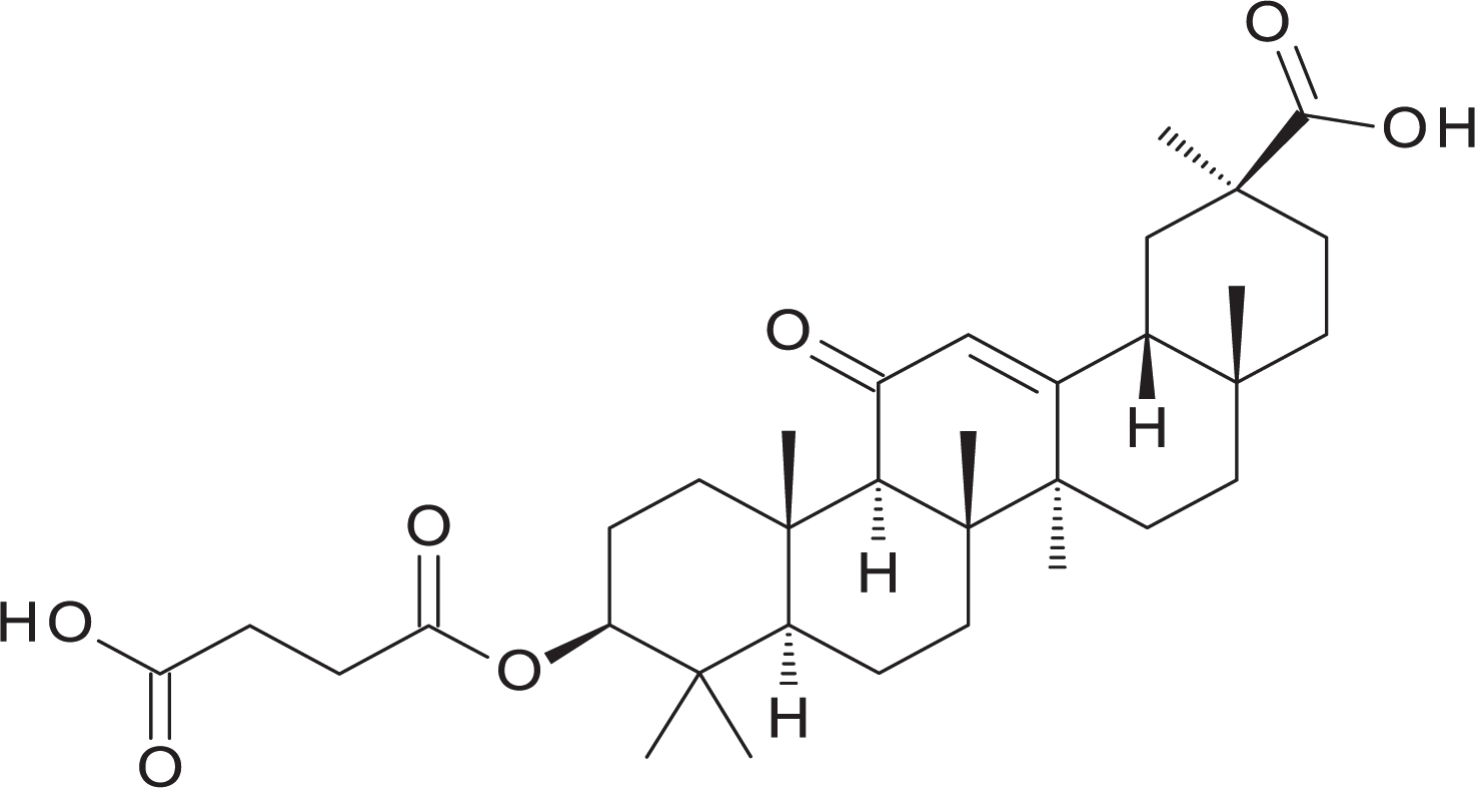
Small molecules bound to FKBP12 in ESI-MS. P represents FKBP12 apoprotein and P:L protein in complex with a ligand.

### Inhibitors of 11β-HSD1

Out of 26 compounds tested, 4 inhibited in cells and with recombinant human 11β-HSD1. IC_50_ values for the SPA cell-based reductase assay and K_i_^app^ for the fluorescent recombinant protein dehydrogenase assay are shown in Table 2. Structures of compounds 5–8 are available in S4 Fig. A compound was classified as a hit if it inhibited 11β-HSD1 by at least 40% in the cell-based assay with a concentration of 100 μM inhibitor and 25% inhibition in the recombinant protein assay. The fluorescence assay is less sensitive to inhibition due to the partial presence of carbenoxolone in the enzyme binding site as an artifact of the purification procedure. This is reflected in lower inhibition by the same compounds in this assay, but may also be due to differences in the ability of each compound to inhibit either the ‘reductase’ or ‘dehydrogenase’ forms of the enzyme.

**Table 2:** Binding data for 11β-HSD1.

**Compound ID**	**IUPAC name**	SPA* IC_50_ (μM)	**Fluoresc.^#^ K_i_^app^ (μM)**
**5**	(1E,4E)-1,5-bis(3,4-dimethoxyphenyl)penta-1,4-dien-3-one	11.3±2.6	142 ± 66
**6**	Dihydroxyphenyl)-2-oxoethyl]sulfanyl}-1H-tetrazol-1-yl)benzamide	9.6±13.5	47 ± 13
**7**	1-(3,4-dihydroxyphenyl)-2-[(6-methoxy-1H-benzimidazol-2-yl)sulfanyl]ethanone	7.9±3.0	248 ± 73
**8**	1-(4-methoxyphenyl)-2-(2-pyridylsulfanyl)ethanone	0.067±0.003	26 ± 7

Molecules 5, 6, 7 and 8 were retrieved using carbenoxolone as a query molecule supplied to the UFSRAT technique and screened against the EDULISS database. Binding of the top hits was tested using a scintillation proximity assay (SPA) and fluorescence assay (Fluoresc).

* HEK-293 cells transfected with human 11β-HSD1

# Recombinant 11β-HSD1

### Performance comparison of UFSRAT with USR using the DUD-E dataset

Comparing UFSRAT against USR in the recall of active molecules within the top 0.5%, 1%, 2% and 5% of scores shows that UFSRAT consistently outperforms USR when enrichment is averaged across the DUD-E targets. At the 0.5% level, average enrichments are 1.99 (USR) and 3.83 (UFSRAT), UFSRAT providing greater enrichment over USR for 75 out of 102 protein targets. At the 1% level, average enrichments are 1.70 (USR) and 2.87 (UFSRAT), UFSRAT providing greater enrichment over USR for 70 out of 102 protein targets. At the 2% level, average enrichments are 1.41 (USR) and 2.27 (UFSRAT), UFSRAT providing greater enrichment over USR for 67 out of 102 protein targets. Finally, at the 5% level, average enrichments are 1.09 (USR) and 1.22 (UFSRAT), UFSRAT providing greater enrichment over USR for 68 out of 102 protein targets. For individual proteins in the DUD-E dataset, the largest difference in enrichment when UFSRAT is outperformed is 5.6 for USR and 2.1 for UFSRAT, occurring at the 0.5% level. The largest difference in enrichment when UFSRAT outperforms USR is 33.7 for UFSRAT and 13.8 for USR at the 0.5% level. It is interesting to note that whilst UFSRAT outperformed USR, both 3D techniques were greatly outperformed by ECFP4 in this test. ECFP4 displaying on average, 8.9-fold enrichment at the 0.5% level, 11.9-fold at the 1% level, 7.7-fold at the 2% level, and 4-fold at the 5% level. However, whilst ECFP4 performs superbly in this test, its performance can be accredited to the high degree of common substructures shared by DUD-E actives, and absent amongst decoys. The nature by which ECFP and other fingerprint methods operate are less amenable than 3D techniques to radical scaffold hopping to the degree demonstrated by UFSRAT in the discovery 11β-HSD1 actives. Exhaustive results for the recall of actives are available in the supporting data accompanying this report (see S1 Fig. and S1–S6 Tables).

## Discussion

The nature of UFSRAT as a ligand-based molecular similarity technique enables ligand discovery whilst bypassing the need for any detailed structural information on the target. The 3D conformation of a substrate or inhibitor indirectly encodes 3D and electrostatic properties of the active form of the receptor. UFSRAT has an advantage over 1D and 2D-based molecular similarity techniques, describing a complimentary shape to the binding pocket, something which is not captured using 1D or 2D techniques. UFSRAT is also not prescriptive of bond order, specific sub-structures or molecular orientation but does take into account the electropotential and therefore has the ability to discover bioisosteres, strengthening scaffold hopping abilities as already demonstrated for 11β-HSD1.

Defining the similarity between two molecules in an objective way is a non-trivial problem. Validating the success of a similarity program is difficult due to the absence of any absolute metrics of similarity. However, performance comparison using the DUD-E dataset clearly shows enhanced enrichment compared to the original USR method.

Comparison of query and candidate molecules by UFSRAT is sensitive to the conformation of the molecules in question and one might therefore infer that the program would have most success in biological assays if the receptor bound conformation of the query is known. In this study UFSRAT has selected biologically active compounds from a query structure with known conformation derived from a protein-ligand complex and a query where we have generated a low energy conformer from a 2D representation. The pre-calculation of descriptors by UFSRAT means that it is possible to place descriptors for more than one low energy conformation in the database of candidate compounds and also test multiple conformations of a query, thus reducing the chances of missing a hit because virtual molecules were not in biologically relevant conformations.

In this study UFSRAT retrieved the highest affinity hits for 11β-HSD1. For this target the correct biological conformation of the query carbenoxolone was available from crystallographic data. Interestingly, the top virtual hits were non-steroidal but did show similarity to known 11β-HSD1 inhibitors demonstrating the ability of UFSRAT to scaffold hop. By eye, carbenoxolone and all identified 11β-HSD1 active molecules are radically different however, a key pattern of atoms involved in hydrogen bonding is preserved when examined in 3D (see S2 Fig.). Also, a survey of 11β-HSD1 non-steroidal inhibitors has shown that the majority share a central group or linker containing an atom that can act as a hydrogen bond acceptor, typically oxygen, flanked on either side by lipophilic groups [41]. This molecular arrangement is observed with all the hit compounds in this study. Compound **6** contains a linker with a 1,5-sulphanyl 1*H*-tetrazole moiety. A series of bioactive compounds containing the same 1,5-sulphanyl 1*H*-tetrazole linker have been reported [41]. Compounds **6**, **7** and **8** all share the same linker between the lipophilic groups. The highest affinity hit is compound **8** which showed low nanomolar affinity IC_50_ in a cell based assay.

Compounds selected by UFSRAT that bind to FKBP12 with mid-micromolar affinity are similar to the query compounds in that they share two ring systems joined by a linker containing a motif that has the potential to form hydrogen-bonding interactions with hot-spot residues in the active site. The affinity of the query compounds Q2 (15 μM) and Q3 (95 μM) (see Fig. 4) for FKBP12 show that small changes to the linker have a significant affect on affinity. Stebbins reports a series of analogues of Q1 (Fig. 4); substituting—S- with—S0_2_-, -0- or—CH_2_- all result in a reduction in affinity [31]. The biophysical techniques used to assess binding all have the advantage of detecting low affinity ligands that have low solubility in solution. However, as measurements are carried close to 50°C it is likely, in the majority of cases, that the affinity would be higher at 25°C.

## Conclusion

UFSRAT has been shown to be an efficient algorithm for selecting bioactive molecules from large databases. Compounds have been identified that bind to two clinically relevant drug targets FKBP12 and 11β-HSD1. A key feature of the results is that the compounds selected by UFSRAT do not have the same scaffold as query molecule, thus demonstrating the scaffold hopping capabilities of USFRAT.

## Supporting Information

S1 Fig USR and UFSRAT enrichment rates for the recall of actives within the top n% of scoring molecules using the DUDE dataset. (TIF)

S2 Fig Carbenoxolone (top) and 11β-HSD1 active compound #8 (lower) with key hydrogen bond acceptor patter highlighted with approximate moments to center shown. (TIF)

S3 Fig 2D structures of compounds 1–4 and Q2. (TIF)

S4 Fig 2D structures of compounds 5–8. (TIF)

S1 Table Performance comparison statistics for enrichment in recall of active compounds from the DUDE dataset. (DOCX)

S2 Table DUD-E profiling of USR and UFSRAT at the 0.5% level. (DOCX)

S3 Table DUD-E profiling of USR and UFSRAT at the 1% level. (DOCX)

S4 Table DUD-E profiling of USR and UFSRAT at the 2% level. (DOCX)

S5 Table DUD-E profiling of USR and UFSRAT at the 5% level. (DOCX)

S6 Table DUD-E profiling of ECFP4 at the 0.5, 1, 2 and 5% levels. (DOCX)

## References

[1] Bender A, Glen RC (2004) Molecular similarity: a key technique in molecular informatics. Organic & Biomolecular Chemistry 2: 3204–3218. 10.1039/b409813g 15534697

[2] DiMasi JA (2001) Risks in new drug development: approval success rates for investigational drugs. Clin Pharmacol Ther 69: 297–307. 10.1067/mcp.2001.115446 11371997

[3] DiMasi JA, Hansen RW, Grabowski HG (2003) The price of innovation: new estimates of drug development costs. Journal of Health Economics 22: 151–185. 10.1016/S0167-6296(02)00126-1 12606142

[4] Brown RD, Martin YC (1996) Use of Structure-Activity Data To Compare Structure-Based Clustering Methods and Descriptors for Use in Compound Selection. Journal of Chemical Information and Computer Sciences 36: 572–584. 10.1021/ci9501047

[5] Brown RD, Martin YC (1997) The Information Content of 2D and 3D Structural Descriptors Relevant to Ligand-Receptor Binding. Journal of Chemical Information and Computer Sciences 37: 1–9.

[6] Patterson DE, Cramer RD, Ferguson AM, Clark RD, Weinberger LE (1996) Neighborhood Behavior: A Useful Concept for Validation of” Molecular Diversity” Descriptors. Journal of Medicinal Chemistry 39: 3049–3059. 10.1021/jm960290n 8759626

[7] Sheridan RP (2002) The Most Common Chemical Replacements in Drug-Like Compounds. Journal of Chemical Information and Computer Sciences 42: 103–108. 10.1021/ci0100806 11855973

[8] Sheridan RP (2003) Finding Multiactivity Substructures by Mining Databases of Drug-Like Compounds. Journal of Chemical Information and Computer Sciences 43: 1037–1050. 10.1021/ci030004y 12767163

[9] Martin YC, Kofron JL, Traphagen LM (2002) Do Structurally Similar Molecules Have Similar Biological Activity? Journal of Medicinal Chemistry 45: 4350–4358. 10.1021/jm020155c 12213076

[10] van de Waterbeemd H, Gifford E (2003) ADMET in silico modelling: towards prediction paradise? Nature Reviews Drug Discovery 2: 192–204. 10.1038/nrd1032 12612645

[11] Ballester PJ, Richards WG (2007) Ultrafast shape recognition to search compound databases for similar molecular shapes. J Comput Chem 28: 1711–1723. 10.1002/jcc.20681 17342716

[12] Ballester PJ, Richards WG (2007) Ultrafast shape recognition for similarity search in molecular databases. Proceedings of the Royal Society A: Mathematical, Physical and Engineering Sciences 463: 1307–1321. 10.1098/rspa.2007.1823

[13] Hsin KY, Morgan HP, Shave SR, Hinton AC, Taylor P, et al (2011) EDULISS: a small-molecule database with data-mining and pharmacophore searching capabilities. Nucleic Acids Research 39: D1042–D1048. 10.1093/nar/gkq878 21051336 PMC3013767

[14] Ballester PJ, Westwood I, Laurieri N, Sim E, Richards WG (2010) Prospective virtual screening with Ultrafast Shape Recognition: the identification of novel inhibitors of arylamine N-acetyltransferases. Journal of The Royal Society Interface 7: 335–342. 10.1098/rsif.2009.0170 19586957 PMC2842611

[15] Li H, Huang J, Chen L, Liu X, Chen T, et al (2009) Identification of novel falcipain-2 inhibitors as potential antimalarial agents through structure-based virtual screening. Journal of medicinal chemistry 52: 4936–4940. 10.1021/jm801622x 19586036

[16] Ballester PJ, Mangold M, Howard NI, Robinson RLM, Abell C, et al (2012) Hierarchical virtual screening for the discovery of new molecular scaffolds in antibacterial hit identification. Journal of The Royal Society Interface 9: 3196–3207. 10.1098/rsif.2012.0569 22933186 PMC3481598

[17] Steinbeck C, Hoppe C, Kuhn S, Floris M, Guha R, et al (2006) Recent developments of the chemistry development kit (CDK)-an open-source java library for chemo-and bioinformatics. Current Pharmaceutical Design 12: 2111–2120. 10.2174/138161206777585274 16796559

[18] Patil SP, Ballester PJ, Kerezsi CR (2014) Prospective virtual screening for novel p53-MDM2 inhibitors using ultrafast shape recognition. Journal of computer-aided molecular design 28: 89–97. 10.1007/s10822-014-9732-4 24554192

[19] Schreyer AM, Blundell T (2012) USRCAT: real-time ultrafast shape recognition with pharmacophoric constraints. Journal of cheminformatics 4: 1–12. 10.1186/1758-2946-4-27 23131020 PMC3505738

[20] Harding MW, Galat A, Uehling DE, Schreiber SL (1989) A receptor for the immuno-suppressant FK 506 is a cis–trans peptidyl-prolyl isomerase. Nature 341: 758–760. 10.1038/341758a0 2477715

[21] Steiner JP, Dawson TM, Fotuhi M, Glatt CE, Snowman AM, et al (1992) High brain densities of the immunophilin FKBP colocalized with calcineurin. Nature 358: 584–587. 10.1038/358584a0 1380130

[22] Valentine H, Chen Y, Guo H, McCormick J, Wu Y, et al (2007) Neuroimmunophilin ligands protect cavernous nerves after crush injury in the rat: new experimental paradigms. European urology 51: 1724–1731. 10.1016/j.eururo.2006.11.026 17145129 PMC2682459

[23] Shaw G, Gan J, Zhou YN, Zhi H, Subburaman P, et al (2008) Structure of RapA, a Swi2/Snf2 protein that recycles RNA polymerase during transcription. Structure 16: 1417–1427. 10.1016/j.str.2008.06.012 18786404 PMC2607195

[24] Bujalska IJ, Kumar S, Stewart PM (1997) Does central obesity reflect. The Lancet 349: 1210–1213. 10.1016/S0140-6736(96)11222-8 9130942

[25] Tomlinson JW, Walker EA, Bujalska IJ, Draper N, Lavery GG, et al (2004) 11—Hydroxysteroid dehydrogenase type 1: a tissue-specific regulator of glucocorticoid response. Endocrine Reviews 25: 831 10.1210/er.2003-0031 15466942

[26] Taylor P, Blackburn E, Sheng YG, Harding S, Hsin K (2008) Ligand discovery and virtual screening using the program LIDAEUS. British journal of pharmacology 153: S55–S67. 10.1038/sj.bjp.0707532 18037921 PMC2268042

[27] Wu SY, McNae I, Kontopidis G, McClue SJ, McInnes C, et al (2003) Discovery of a Novel Family of CDK Inhibitors with the Program LIDAEUS: Structural Basis for Ligand-Induced Disordering of the Activation Loop. Structure 11: 399–410. 10.1016/S0969-2126(03)00060-1 12679018

[28] Dagum L, Menon R (1998) OpenMP: an industry standard API for shared-memory programming. Computational Science & Engineering, IEEE 5: 46–55. 10.1109/99.660313

[29] Bierer BE, Mattila PS, Standaert RF, Herzenberg LA, Burakoff SJ, et al (1990) Two distinct signal transmission pathways in T lymphocytes are inhibited by complexes formed between an immunophilin and either FK506 or rapamycin. Proceedings of the National Academy of Sciences 87: 9231–9235. 10.1073/pnas.87.23.9231 PMC55138 2123553

[30] Fulton KF, Jackson SE, Buckle AM (2003) Energetic and structural analysis of the role of tryptophan 59 in FKBP12. Biochemistry 42: 2364–2372. 10.1021/bi020564a 12600203

[31] Stebbins JL, Zhang Z, Chen J, Wu B, Emdadi A, et al (2007) Nuclear magnetic resonance fragment-based identification of novel FKBP12 inhibitors. Journal of medicinal chemistry 50: 6607–6617. 10.1021/jm0707424 18038971

[32] Shave SR, Taylor P, Walkinshaw M, Smith L, Hardy J, et al (2008) Ligand discovery on massively parallel systems. IBM Journal of Research and Development 52: 57 10.1147/rd.521.0057

[33] Lipinski CA, Lombardo F, Dominy BW, Feeney PJ (1997) Experimental and computational approaches to estimate solubility and permeability in drug discovery and development settings. Advanced drug discovery reviews 46: 3–26. 10.1016/S0169-409X(96)00423-1 11259830

[34] Arampatzis S, Kadereit B, Schuster D, Balazs Z, Schweizer RAS, et al (2005) Comparative enzymology of 11beta-hydroxysteroid dehydrogenase type 1 from six species. J Neuroimmunol 157: 126–132. 10.1677/jme.1.01736 16087724

[35] Wear MA, Patterson A, Walkinshaw MD (2007) A kinetically trapped intermediate of FK506 binding protein forms *in vitro*: Chaperone machinery dominates protein folding *in vivo* . Protein expression and purification 51: 80–95. 10.1016/j.pep.2006.06.019 16908189

[36] Adie J (2010) PhD Theis; Structure-based drug design of 11β-hydroxysteroid dehydrogenase type 1 inhibitors. University of Edinburgh.

[37] DeLano WL (2002) The PyMOL Molecular Graphics System. DeLano Scientific, San Carlos, CA.

[38] Xing L, Hodgkin E, Liu Q, Sedlock D (2004) Evaluation and application of multiple scoring functions for a virtual screening experiment. Journal of Computer Aided Molecular Design 18: 333–344. 10.1023/B:JCAM.0000047812.39758.ab 15595460

[39] Brandts JF, Lin LN (1990) Study of strong to ultratight protein interactions using differential scanning calorimetry. Biochemistry 29: 6927–6940. 10.1021/bi00481a024 2204424

[40] Tjernberg A, Carnö SS, Oliv F, Benkestock K, Edlund P-O, et al (2004) Determination of dissociation constants for protein-ligand complexes by electrospray ionization mass spectrometry. Analytical chemistry 76: 4325–4331. 10.1021/ac0497914 15283568

[41] Webster SP, Pallin TD (2007) 11ß-Hydroxysteroid dehydrogenase type 1 inhibitors as therapeutic agents. Expert Opinion on Therapeutic Patents 17: 1407 10.1517/13543776.17.12.1407

[42] Mundt S, Solly K, Thieringer R, Hermanowski-Vosatka A (2005) Development and application of a scintillation proximity assay (SPA) for identification of selective inhibitors of 11-hydroxysteroid dehydrogenase type 1. Assay and drug development technologies 3: 367–375. 10.1089/adt.2005.3.367 16180991

[43] Morrison JF (1969) Kinetics of the reversible inhibition of enzyme-catalysed reactions by tight-binding inhibitors. Biochimica et Biophysica Acta (BBA)-Enzymology 185: 269–286. 10.1016/0005-2744(69)90420-3 4980133

[44] Mysinger MM, Carchia M, Irwin JJ, Shoichet BK (2012) Directory of useful decoys, enhanced (DUD-E): better ligands and decoys for better benchmarking. Journal of medicinal chemistry 55: 6582–6594. 10.1021/jm300687e 22716043 PMC3405771

[45] Rogers D, Hahn M (2010) Extended-connectivity fingerprints. Journal of chemical information and modeling 50: 742–754. 10.1021/ci100050t 20426451

[46] Landrum G (2006) RDKit: Open-source cheminformatics. Available: http://www.rdkit.org. Accessed 2013 Jul 7.

