# Correction: UFSRAT: Ultra-Fast Shape Recognition with Atom Types –The Discovery of Novel Bioactive Small Molecular Scaffolds for FKBP12 and 11βHSD1

**DOI:** 10.1371/journal.pone.0122658

**Published:** 2015-03-13

**Authors:** The PLOS ONE Staff

The figure legends for Figs. 5 and 6 are incorrectly switched. Please view the correctly ordered figure legends below.

**Fig 5:**
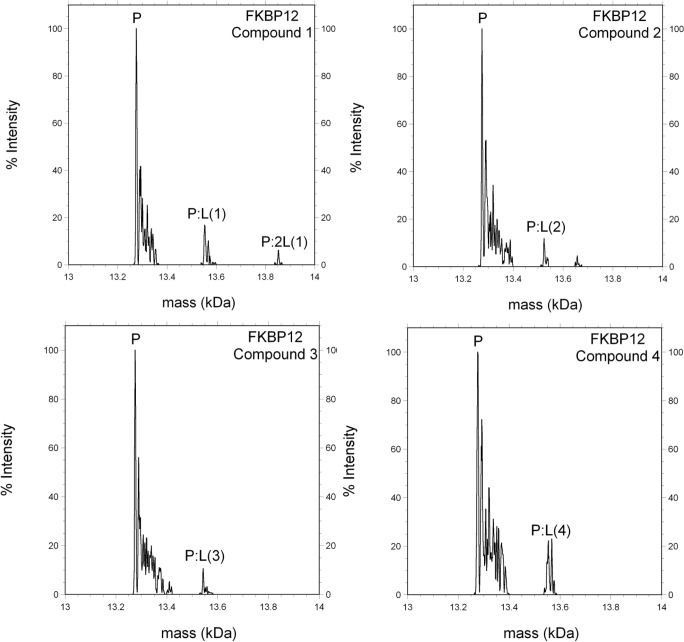
Small molecules bound to FKBP12 in ESI-MS.

**Fig 6:**
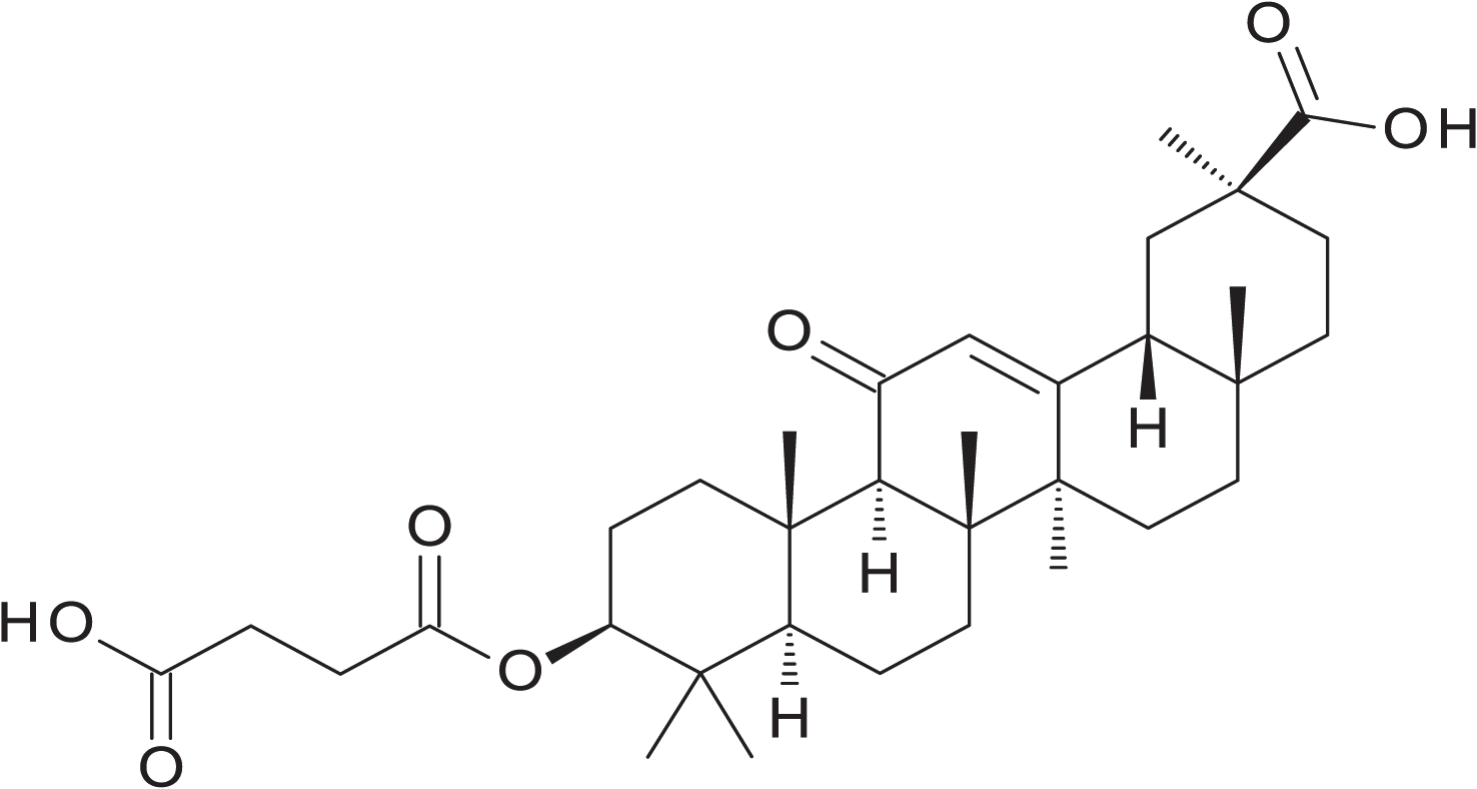
Carbenoxolone, 20nM inhibitor of 11β-HSD1.
