# Supplementary figures and images for: UFSRAT: Ultra-Fast Shape Recognition with Atom Types –The Discovery of Novel Bioactive Small Molecular Scaffolds for FKBP12 and 11βHSD1
Source: PLoS One. 2015 Feb 6;10(2):e0116570. doi: 10.1371/journal.pone.0116570 (PMC4319890; doi:10.1371/journal.pone.0116570)

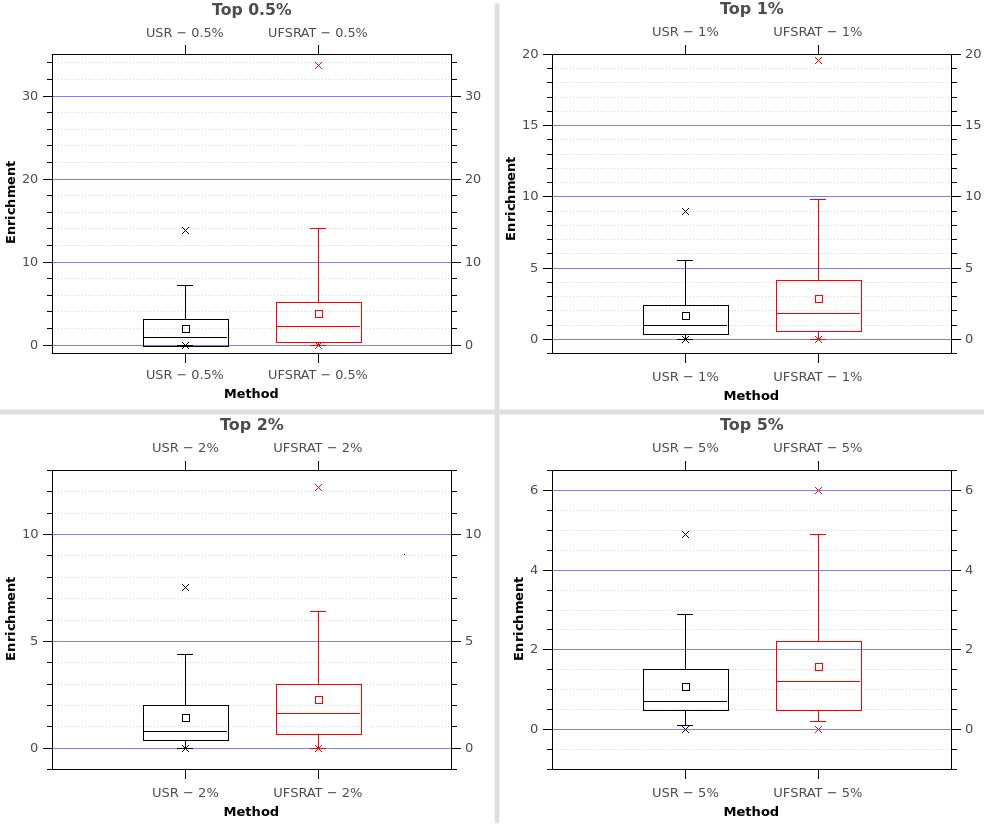

Supplement: S1 Fig — (TIF) [file pone.0116570.s001.tif]

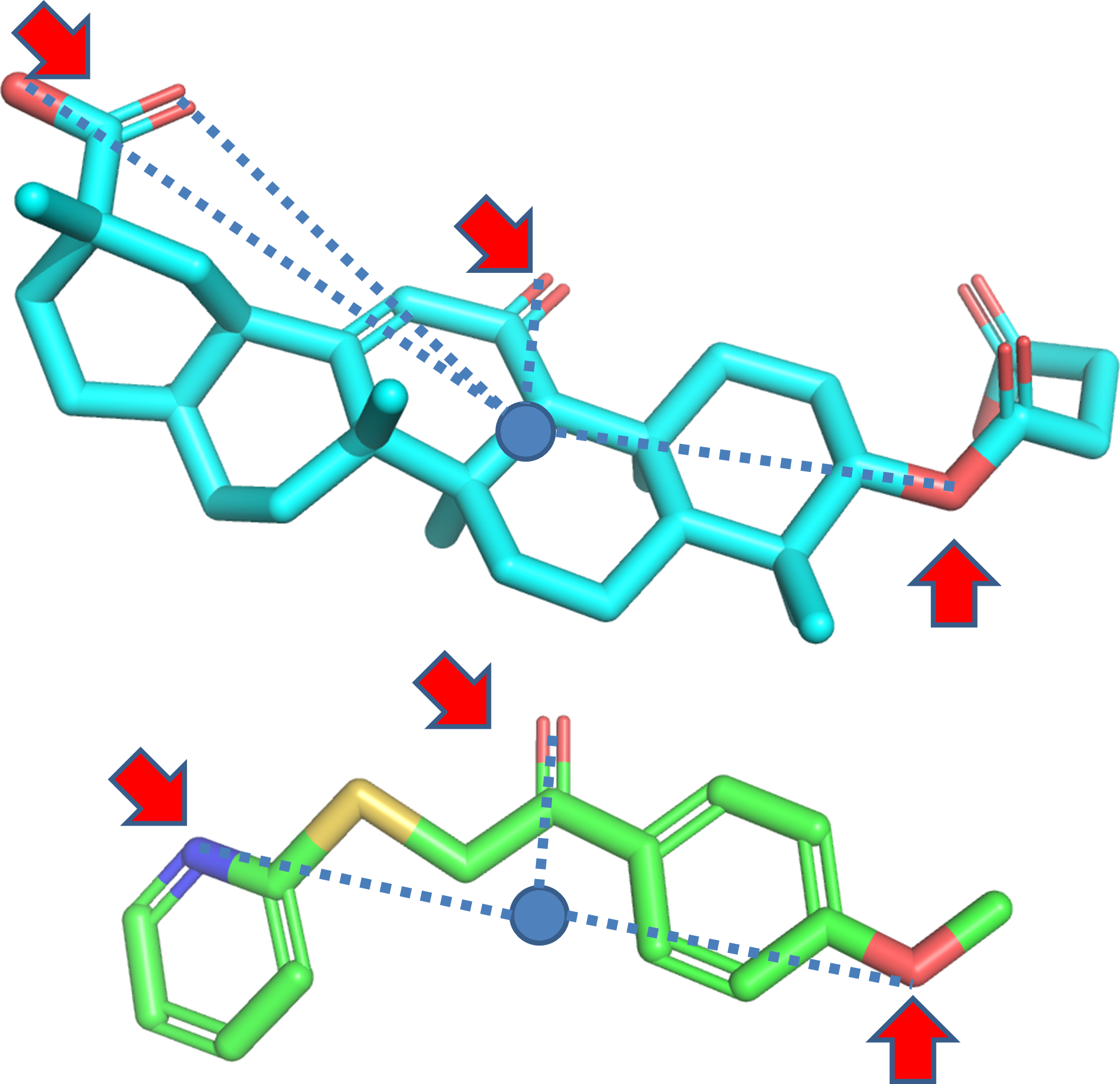

Supplement: S2 Fig — (TIF) [file pone.0116570.s002.tif]

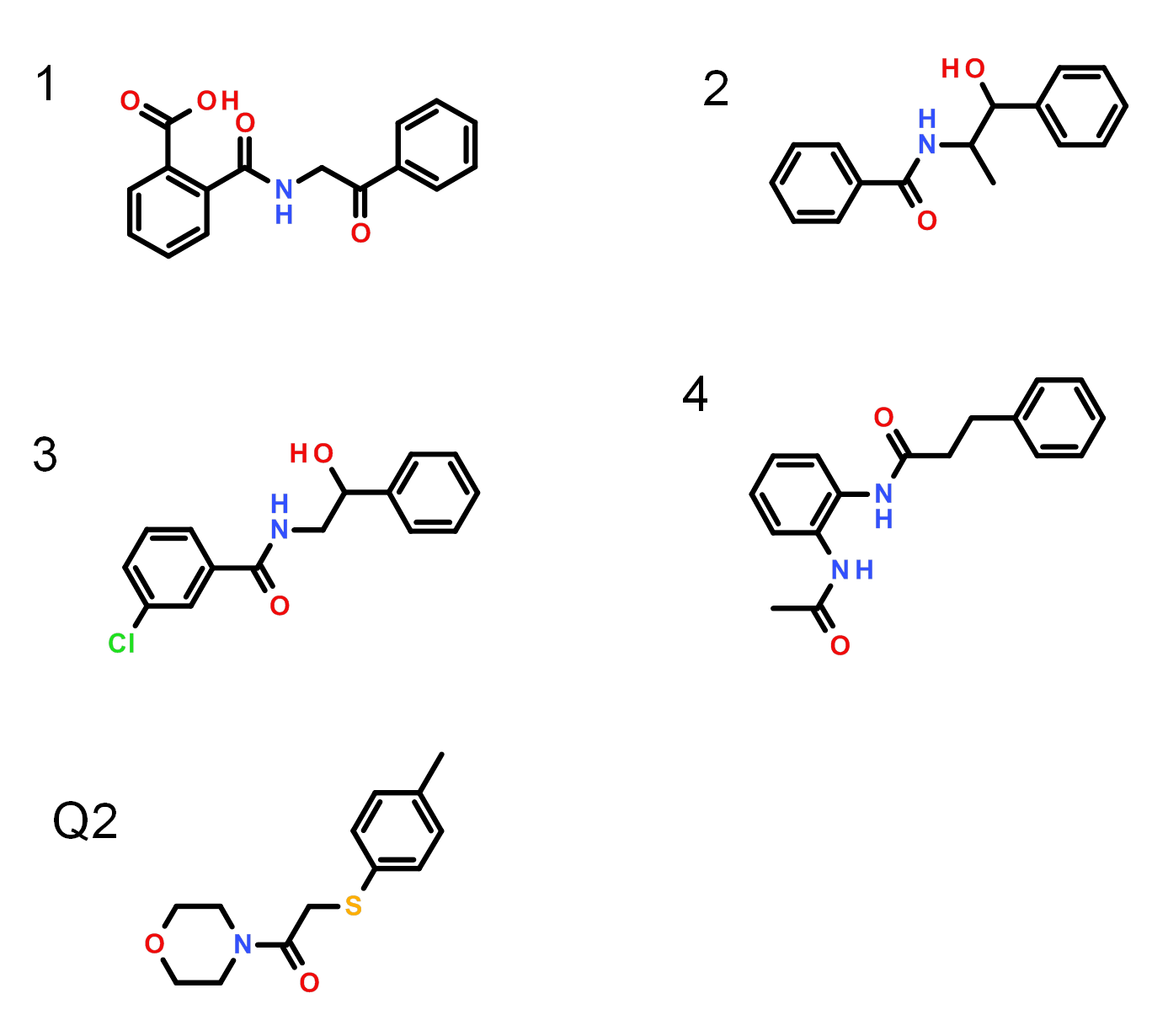

Supplement: S3 Fig — (TIF) [file pone.0116570.s003.tif]

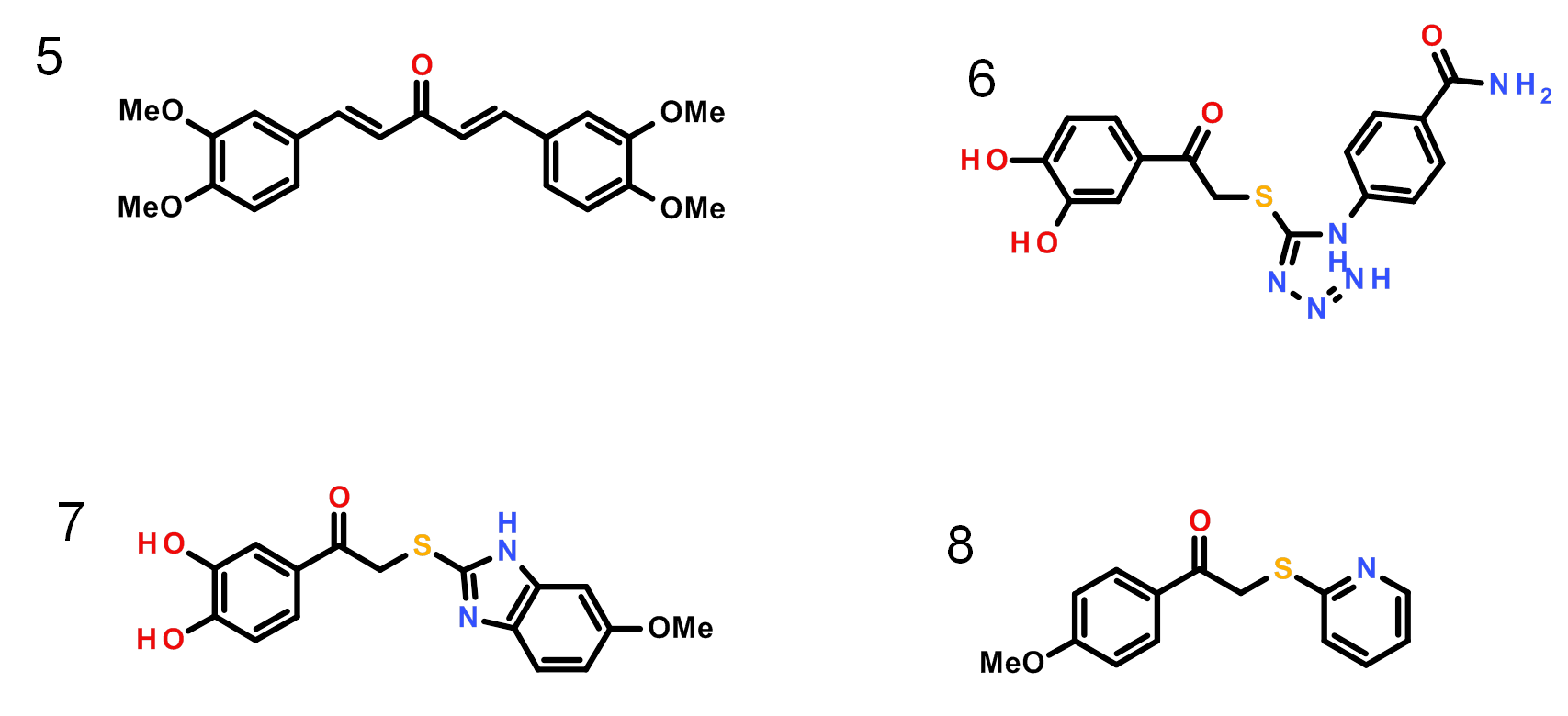

Supplement: S4 Fig — (TIF) [file pone.0116570.s004.tif]
