# Supplementary material for: UFSRAT: Ultra-Fast Shape Recognition with Atom Types –The Discovery of Novel Bioactive Small Molecular Scaffolds for FKBP12 and 11βHSD1
Source: PLoS One. 2015 Feb 6;10(2):e0116570. doi: 10.1371/journal.pone.0116570 (PMC4319890; doi:10.1371/journal.pone.0116570)
Supplement: S1 Table — (DOCX) [file pone.0116570.s005.docx]

Table S1 - Performance comparison statistics for enrichment in recall of active compounds from the DUDE dataset

| Enrichment at level | 0.5% | | | 1% | | | 2% | | | 5% | | |
| --- | --- | --- | --- | --- | --- | --- | --- | --- | --- | --- | --- | --- |
| Technique | USR | UFSRAT | ECFP4 | USR | UFSRAT | ECFP4 | USR | UFSRAT | ECFP4 | USR | UFSRAT | ECFP4 |
| Max | 13.8 | 33.7 | 45.6 | 9.0 | 19.6 | 45.3 | 7.5 | 12.2 | 32.5 | 4.9 | 6.0 | 13.8 |
| Average | 2.0 | 3.8 | 8.9 | 1.7 | 2.9 | 11.9 | 1.4 | 2.3 | 7.7 | 1.1 | 1.6 | 4.0 |
| Quartile 1 | 0.0 | 0.4 | 0.8 | 0.4 | 0.6 | 3.4 | 0.4 | 0.7 | 2.1 | 0.5 | 0.5 | 1.4 |
| Quartile 2 | 0.9 | 2.2 | 3.4 | 1.0 | 1.8 | 9.9 | 0.8 | 1.6 | 6.1 | 0.7 | 1.2 | 3.5 |
| Quartile 3 | 3.1 | 5.2 | 12.6 | 2.4 | 4.1 | 16.6 | 2.0 | 3.0 | 11.9 | 1.5 | 2.2 | 6.4 |
