# Supplementary material for: UFSRAT: Ultra-Fast Shape Recognition with Atom Types –The Discovery of Novel Bioactive Small Molecular Scaffolds for FKBP12 and 11βHSD1
Source: PLoS One. 2015 Feb 6;10(2):e0116570. doi: 10.1371/journal.pone.0116570 (PMC4319890; doi:10.1371/journal.pone.0116570)
Supplement: S2 Table — (DOCX) [file pone.0116570.s006.docx]

Table S2 - DUD-E profiling of USR and UFSRAT at the 0.5% level

| Target | Method | Hits | Possible hits | Success_rate | Library_actives | Library_total | Library_actives_proportion | Enrichment |
| --- | --- | --- | --- | --- | --- | --- | --- | --- |
| aa2ar | usr | 3 | 164 | 1.83% | 844 | 32908 | 2.56% | 0.7 |
| aa2ar | ufsrat | 22 | 164 | 13.41% | 844 | 32908 | 2.56% | 5.2 |
| abl1 | usr | 1 | 55 | 1.82% | 295 | 11180 | 2.64% | 0.7 |
| abl1 | ufsrat | 4 | 55 | 7.27% | 295 | 11180 | 2.64% | 2.8 |
| ace | usr | 2 | 89 | 2.25% | 808 | 17952 | 4.50% | 0.5 |
| ace | ufsrat | 12 | 89 | 13.48% | 808 | 17952 | 4.50% | 3 |
| aces | usr | 1 | 135 | 0.74% | 664 | 27037 | 2.46% | 0.3 |
| aces | ufsrat | 2 | 135 | 1.48% | 664 | 27037 | 2.46% | 0.6 |
| ada | usr | 0 | 28 | 0.00% | 262 | 5734 | 4.57% | 0 |
| ada | ufsrat | 4 | 28 | 14.29% | 262 | 5734 | 4.57% | 3.1 |
| ada17 | usr | 6 | 188 | 3.19% | 959 | 37606 | 2.55% | 1.3 |
| ada17 | ufsrat | 0 | 188 | 0.00% | 959 | 37606 | 2.55% | 0 |
| adrb1 | usr | 1 | 82 | 1.22% | 458 | 16416 | 2.79% | 0.4 |
| adrb1 | ufsrat | 0 | 82 | 0.00% | 458 | 16416 | 2.79% | 0 |
| adrb2 | usr | 2 | 78 | 2.56% | 447 | 15702 | 2.85% | 0.9 |
| adrb2 | ufsrat | 1 | 78 | 1.28% | 447 | 15702 | 2.85% | 0.4 |
| akt1 | usr | 2 | 84 | 2.38% | 423 | 16999 | 2.49% | 1 |
| akt1 | ufsrat | 1 | 84 | 1.19% | 423 | 16999 | 2.49% | 0.5 |
| akt2 | usr | 0 | 35 | 0.00% | 190 | 7142 | 2.66% | 0 |
| akt2 | ufsrat | 6 | 35 | 17.14% | 190 | 7142 | 2.66% | 6.4 |
| aldr | usr | 0 | 46 | 0.00% | 220 | 9356 | 2.35% | 0 |
| aldr | ufsrat | 0 | 46 | 0.00% | 220 | 9356 | 2.35% | 0 |
| ampc | usr | 0 | 14 | 0.00% | 62 | 2964 | 2.09% | 0 |
| ampc | ufsrat | 3 | 14 | 21.43% | 62 | 2964 | 2.09% | 10.3 |
| andr | usr | 10 | 75 | 13.33% | 523 | 15026 | 3.48% | 3.8 |
| andr | ufsrat | 22 | 75 | 29.33% | 523 | 15026 | 3.48% | 8.4 |
| aofb | usr | 1 | 35 | 2.86% | 168 | 7099 | 2.37% | 1.2 |
| aofb | ufsrat | 2 | 35 | 5.71% | 168 | 7099 | 2.37% | 2.4 |
| bace1 | usr | 2 | 93 | 2.15% | 485 | 18706 | 2.59% | 0.8 |
| bace1 | ufsrat | 3 | 93 | 3.23% | 485 | 18706 | 2.59% | 1.2 |
| braf | usr | 5 | 51 | 9.80% | 251 | 10349 | 2.43% | 4 |
| braf | ufsrat | 3 | 51 | 5.88% | 251 | 10349 | 2.43% | 2.4 |
| cah2 | usr | 2 | 162 | 1.23% | 835 | 32545 | 2.57% | 0.5 |
| cah2 | ufsrat | 9 | 162 | 5.56% | 835 | 32545 | 2.57% | 2.2 |
| casp3 | usr | 1 | 55 | 1.82% | 350 | 11172 | 3.13% | 0.6 |
| casp3 | ufsrat | 6 | 55 | 10.91% | 350 | 11172 | 3.13% | 3.5 |
| cdk2 | usr | 4 | 145 | 2.76% | 798 | 29126 | 2.74% | 1 |
| cdk2 | ufsrat | 9 | 145 | 6.21% | 798 | 29126 | 2.74% | 2.3 |
| comt | usr | 1 | 20 | 5.00% | 86 | 4012 | 2.14% | 2.3 |
| comt | ufsrat | 6 | 20 | 30.00% | 86 | 4012 | 2.14% | 14 |
| cp2c9 | usr | 0 | 38 | 0.00% | 183 | 7757 | 2.36% | 0 |
| cp2c9 | ufsrat | 1 | 38 | 2.63% | 183 | 7757 | 2.36% | 1.1 |
| cp3a4 | usr | 1 | 61 | 1.64% | 363 | 12303 | 2.95% | 0.6 |
| cp3a4 | ufsrat | 1 | 61 | 1.64% | 363 | 12303 | 2.95% | 0.6 |
| csf1r | usr | 8 | 63 | 12.70% | 286 | 12720 | 2.25% | 5.6 |
| csf1r | ufsrat | 3 | 63 | 4.76% | 286 | 12720 | 2.25% | 2.1 |
| cxcr4 | usr | 2 | 17 | 11.76% | 122 | 3536 | 3.45% | 3.4 |
| cxcr4 | ufsrat | 3 | 17 | 17.65% | 122 | 3536 | 3.45% | 5.1 |
| def | usr | 2 | 29 | 6.90% | 161 | 5899 | 2.73% | 2.5 |
| def | ufsrat | 5 | 29 | 17.24% | 161 | 5899 | 2.73% | 6.3 |
| dhi1 | usr | 2 | 100 | 2.00% | 519 | 20142 | 2.58% | 0.8 |
| dhi1 | ufsrat | 1 | 100 | 1.00% | 519 | 20142 | 2.58% | 0.4 |
| dpp4 | usr | 11 | 212 | 5.19% | 1079 | 42452 | 2.54% | 2 |
| dpp4 | ufsrat | 4 | 212 | 1.89% | 1079 | 42452 | 2.54% | 0.7 |
| drd3 | usr | 3 | 175 | 1.71% | 877 | 35065 | 2.50% | 0.7 |
| drd3 | ufsrat | 1 | 175 | 0.57% | 877 | 35065 | 2.50% | 0.2 |
| dyr | usr | 0 | 89 | 0.00% | 566 | 17950 | 3.15% | 0 |
| dyr | ufsrat | 5 | 89 | 5.62% | 566 | 17950 | 3.15% | 1.8 |
| egfr | usr | 1 | 181 | 0.55% | 832 | 36274 | 2.29% | 0.2 |
| egfr | ufsrat | 0 | 181 | 0.00% | 832 | 36274 | 2.29% | 0 |
| esr1 | usr | 12 | 107 | 11.21% | 627 | 21445 | 2.92% | 3.8 |
| esr1 | ufsrat | 23 | 107 | 21.50% | 627 | 21445 | 2.92% | 7.4 |
| esr2 | usr | 11 | 104 | 10.58% | 595 | 20908 | 2.85% | 3.7 |
| esr2 | ufsrat | 19 | 104 | 18.27% | 595 | 20908 | 2.85% | 6.4 |
| fa10 | usr | 16 | 106 | 15.09% | 792 | 21209 | 3.73% | 4 |
| fa10 | ufsrat | 11 | 106 | 10.38% | 792 | 21209 | 3.73% | 2.8 |
| fa7 | usr | 2 | 32 | 6.25% | 185 | 6487 | 2.85% | 2.2 |
| fa7 | ufsrat | 3 | 32 | 9.38% | 185 | 6487 | 2.85% | 3.3 |
| fabp4 | usr | 0 | 14 | 0.00% | 57 | 2912 | 1.96% | 0 |
| fabp4 | ufsrat | 0 | 14 | 0.00% | 57 | 2912 | 1.96% | 0 |
| fak1 | usr | 0 | 27 | 0.00% | 114 | 5516 | 2.07% | 0 |
| fak1 | ufsrat | 1 | 27 | 3.70% | 114 | 5516 | 2.07% | 1.8 |
| fkb1a | usr | 0 | 30 | 0.00% | 273 | 6105 | 4.47% | 0 |
| fkb1a | ufsrat | 2 | 30 | 6.67% | 273 | 6105 | 4.47% | 1.5 |
| fnta | usr | 8 | 268 | 2.99% | 1692 | 53741 | 3.15% | 0.9 |
| fnta | ufsrat | 3 | 268 | 1.12% | 1692 | 53741 | 3.15% | 0.4 |
| fpps | usr | 7 | 46 | 15.22% | 213 | 9228 | 2.31% | 6.6 |
| fpps | ufsrat | 22 | 46 | 47.83% | 213 | 9228 | 2.31% | 20.7 |
| gcr | usr | 1 | 78 | 1.28% | 563 | 15748 | 3.58% | 0.4 |
| gcr | ufsrat | 2 | 78 | 2.56% | 563 | 15748 | 3.58% | 0.7 |
| glcm | usr | 1 | 20 | 5.00% | 313 | 4150 | 7.54% | 0.7 |
| glcm | ufsrat | 5 | 20 | 25.00% | 313 | 4150 | 7.54% | 3.3 |
| gria2 | usr | 8 | 61 | 13.11% | 297 | 12358 | 2.40% | 5.5 |
| gria2 | ufsrat | 2 | 61 | 3.28% | 297 | 12358 | 2.40% | 1.4 |
| grik1 | usr | 0 | 33 | 0.00% | 152 | 6769 | 2.25% | 0 |
| grik1 | ufsrat | 0 | 33 | 0.00% | 152 | 6769 | 2.25% | 0 |
| hdac2 | usr | 0 | 53 | 0.00% | 238 | 10604 | 2.24% | 0 |
| hdac2 | ufsrat | 3 | 53 | 5.66% | 238 | 10604 | 2.24% | 2.5 |
| hdac8 | usr | 0 | 53 | 0.00% | 234 | 10748 | 2.18% | 0 |
| hdac8 | ufsrat | 4 | 53 | 7.55% | 234 | 10748 | 2.18% | 3.5 |
| hivint | usr | 0 | 34 | 0.00% | 211 | 6967 | 3.03% | 0 |
| hivint | ufsrat | 1 | 34 | 2.94% | 211 | 6967 | 3.03% | 1 |
| hivpr | usr | 15 | 188 | 7.98% | 1395 | 37673 | 3.70% | 2.2 |
| hivpr | ufsrat | 7 | 188 | 3.72% | 1395 | 37673 | 3.70% | 1 |
| hivrt | usr | 7 | 98 | 7.14% | 639 | 19773 | 3.23% | 2.2 |
| hivrt | ufsrat | 10 | 98 | 10.20% | 639 | 19773 | 3.23% | 3.2 |
| hmdh | usr | 2 | 45 | 4.44% | 299 | 9183 | 3.26% | 1.4 |
| hmdh | ufsrat | 5 | 45 | 11.11% | 299 | 9183 | 3.26% | 3.4 |
| hs90a | usr | 2 | 25 | 8.00% | 125 | 5067 | 2.47% | 3.2 |
| hs90a | ufsrat | 0 | 25 | 0.00% | 125 | 5067 | 2.47% | 0 |
| hxk4 | usr | 0 | 24 | 0.00% | 127 | 4930 | 2.58% | 0 |
| hxk4 | ufsrat | 7 | 24 | 29.17% | 127 | 4930 | 2.58% | 11.3 |
| igf1r | usr | 0 | 48 | 0.00% | 226 | 9633 | 2.35% | 0 |
| igf1r | ufsrat | 3 | 48 | 6.25% | 226 | 9633 | 2.35% | 2.7 |
| inha | usr | 0 | 11 | 0.00% | 71 | 2389 | 2.97% | 0 |
| inha | ufsrat | 4 | 11 | 36.36% | 71 | 2389 | 2.97% | 12.2 |
| ital | usr | 1 | 44 | 2.27% | 233 | 8923 | 2.61% | 0.9 |
| ital | ufsrat | 6 | 44 | 13.64% | 233 | 8923 | 2.61% | 5.2 |
| jak2 | usr | 5 | 33 | 15.15% | 153 | 6743 | 2.27% | 6.7 |
| jak2 | ufsrat | 4 | 33 | 12.12% | 153 | 6743 | 2.27% | 5.3 |
| kif11 | usr | 0 | 35 | 0.00% | 197 | 7109 | 2.77% | 0 |
| kif11 | ufsrat | 0 | 35 | 0.00% | 197 | 7109 | 2.77% | 0 |
| kit | usr | 1 | 54 | 1.85% | 252 | 10861 | 2.32% | 0.8 |
| kit | ufsrat | 0 | 54 | 0.00% | 252 | 10861 | 2.32% | 0 |
| kith | usr | 1 | 14 | 7.14% | 132 | 2998 | 4.40% | 1.6 |
| kith | ufsrat | 5 | 14 | 35.71% | 132 | 2998 | 4.40% | 8.1 |
| kpcb | usr | 8 | 45 | 17.78% | 248 | 9092 | 2.73% | 6.5 |
| kpcb | ufsrat | 6 | 45 | 13.33% | 248 | 9092 | 2.73% | 4.9 |
| lck | usr | 0 | 142 | 0.00% | 683 | 28539 | 2.39% | 0 |
| lck | ufsrat | 9 | 142 | 6.34% | 683 | 28539 | 2.39% | 2.7 |
| lkha4 | usr | 2 | 48 | 4.17% | 244 | 9721 | 2.51% | 1.7 |
| lkha4 | ufsrat | 1 | 48 | 2.08% | 244 | 9721 | 2.51% | 0.8 |
| mapk2 | usr | 1 | 32 | 3.13% | 206 | 6450 | 3.19% | 1 |
| mapk2 | ufsrat | 0 | 32 | 0.00% | 206 | 6450 | 3.19% | 0 |
| mcr | usr | 3 | 27 | 11.11% | 193 | 5433 | 3.55% | 3.1 |
| mcr | ufsrat | 10 | 27 | 37.04% | 193 | 5433 | 3.55% | 10.4 |
| met | usr | 12 | 58 | 20.69% | 244 | 11677 | 2.09% | 9.9 |
| met | ufsrat | 13 | 58 | 22.41% | 244 | 11677 | 2.09% | 10.7 |
| mk01 | usr | 1 | 23 | 4.35% | 139 | 4767 | 2.92% | 1.5 |
| mk01 | ufsrat | 1 | 23 | 4.35% | 139 | 4767 | 2.92% | 1.5 |
| mk10 | usr | 1 | 34 | 2.94% | 186 | 6900 | 2.70% | 1.1 |
| mk10 | ufsrat | 0 | 34 | 0.00% | 186 | 6900 | 2.70% | 0 |
| mk14 | usr | 7 | 186 | 3.76% | 915 | 37347 | 2.45% | 1.5 |
| mk14 | ufsrat | 2 | 186 | 1.08% | 915 | 37347 | 2.45% | 0.4 |
| mmp13 | usr | 5 | 195 | 2.56% | 1038 | 39046 | 2.66% | 1 |
| mmp13 | ufsrat | 10 | 195 | 5.13% | 1038 | 39046 | 2.66% | 1.9 |
| mp2k1 | usr | 9 | 42 | 21.43% | 242 | 8483 | 2.85% | 7.5 |
| mp2k1 | ufsrat | 11 | 42 | 26.19% | 242 | 8483 | 2.85% | 9.2 |
| nos1 | usr | 0 | 41 | 0.00% | 234 | 8307 | 2.82% | 0 |
| nos1 | ufsrat | 0 | 41 | 0.00% | 234 | 8307 | 2.82% | 0 |
| nram | usr | 12 | 32 | 37.50% | 222 | 6449 | 3.44% | 10.9 |
| nram | ufsrat | 17 | 32 | 53.13% | 222 | 6449 | 3.44% | 15.4 |
| pa2ga | usr | 0 | 26 | 0.00% | 127 | 5343 | 2.38% | 0 |
| pa2ga | ufsrat | 2 | 26 | 7.69% | 127 | 5343 | 2.38% | 3.2 |
| parp1 | usr | 14 | 155 | 9.03% | 742 | 31171 | 2.38% | 3.8 |
| parp1 | ufsrat | 5 | 155 | 3.23% | 742 | 31171 | 2.38% | 1.4 |
| pde5a | usr | 7 | 142 | 4.93% | 706 | 28532 | 2.47% | 2 |
| pde5a | ufsrat | 10 | 142 | 7.04% | 706 | 28532 | 2.47% | 2.9 |
| pgh1 | usr | 1 | 55 | 1.82% | 251 | 11193 | 2.24% | 0.8 |
| pgh1 | ufsrat | 1 | 55 | 1.82% | 251 | 11193 | 2.24% | 0.8 |
| pgh2 | usr | 24 | 119 | 20.17% | 531 | 23936 | 2.22% | 9.1 |
| pgh2 | ufsrat | 41 | 119 | 34.45% | 531 | 23936 | 2.22% | 15.5 |
| plk1 | usr | 2 | 35 | 5.71% | 155 | 7034 | 2.20% | 2.6 |
| plk1 | ufsrat | 0 | 35 | 0.00% | 155 | 7034 | 2.20% | 0 |
| pnph | usr | 4 | 36 | 11.11% | 233 | 7249 | 3.21% | 3.5 |
| pnph | ufsrat | 8 | 36 | 22.22% | 233 | 7249 | 3.21% | 6.9 |
| ppara | usr | 16 | 101 | 15.84% | 544 | 20375 | 2.67% | 5.9 |
| ppara | ufsrat | 8 | 101 | 7.92% | 544 | 20375 | 2.67% | 3 |
| ppard | usr | 0 | 67 | 0.00% | 288 | 13520 | 2.13% | 0 |
| ppard | ufsrat | 0 | 67 | 0.00% | 288 | 13520 | 2.13% | 0 |
| pparg | usr | 4 | 132 | 3.03% | 723 | 26590 | 2.72% | 1.1 |
| pparg | ufsrat | 4 | 132 | 3.03% | 723 | 26590 | 2.72% | 1.1 |
| prgr | usr | 16 | 81 | 19.75% | 444 | 16258 | 2.73% | 7.2 |
| prgr | ufsrat | 17 | 81 | 20.99% | 444 | 16258 | 2.73% | 7.7 |
| ptn1 | usr | 1 | 38 | 2.63% | 225 | 7658 | 2.94% | 0.9 |
| ptn1 | ufsrat | 1 | 38 | 2.63% | 225 | 7658 | 2.94% | 0.9 |
| pur2 | usr | 0 | 14 | 0.00% | 201 | 2926 | 6.87% | 0 |
| pur2 | ufsrat | 0 | 14 | 0.00% | 201 | 2926 | 6.87% | 0 |
| pygm | usr | 2 | 20 | 10.00% | 114 | 4159 | 2.74% | 3.6 |
| pygm | ufsrat | 8 | 20 | 40.00% | 114 | 4159 | 2.74% | 14.6 |
| pyrd | usr | 9 | 33 | 27.27% | 134 | 6782 | 1.98% | 13.8 |
| pyrd | ufsrat | 22 | 33 | 66.67% | 134 | 6782 | 1.98% | 33.7 |
| reni | usr | 1 | 36 | 2.78% | 387 | 7371 | 5.25% | 0.5 |
| reni | ufsrat | 2 | 36 | 5.56% | 387 | 7371 | 5.25% | 1.1 |
| rock1 | usr | 0 | 32 | 0.00% | 203 | 6580 | 3.09% | 0 |
| rock1 | ufsrat | 0 | 32 | 0.00% | 203 | 6580 | 3.09% | 0 |
| rxra | usr | 1 | 39 | 2.56% | 162 | 7869 | 2.06% | 1.2 |
| rxra | ufsrat | 3 | 39 | 7.69% | 162 | 7869 | 2.06% | 3.7 |
| sahh | usr | 3 | 18 | 16.67% | 190 | 3673 | 5.17% | 3.2 |
| sahh | ufsrat | 5 | 18 | 27.78% | 190 | 3673 | 5.17% | 5.4 |
| src | usr | 1 | 178 | 0.56% | 831 | 35790 | 2.32% | 0.2 |
| src | ufsrat | 12 | 178 | 6.74% | 831 | 35790 | 2.32% | 2.9 |
| tgfr1 | usr | 8 | 44 | 18.18% | 281 | 8958 | 3.14% | 5.8 |
| tgfr1 | ufsrat | 13 | 44 | 29.55% | 281 | 8958 | 3.14% | 9.4 |
| thb | usr | 0 | 39 | 0.00% | 168 | 7821 | 2.15% | 0 |
| thb | ufsrat | 9 | 39 | 23.08% | 168 | 7821 | 2.15% | 10.7 |
| thrb | usr | 1 | 140 | 0.71% | 861 | 28182 | 3.06% | 0.2 |
| thrb | ufsrat | 1 | 140 | 0.71% | 861 | 28182 | 3.06% | 0.2 |
| try1 | usr | 8 | 134 | 5.97% | 758 | 26977 | 2.81% | 2.1 |
| try1 | ufsrat | 1 | 134 | 0.75% | 758 | 26977 | 2.81% | 0.3 |
| tryb1 | usr | 0 | 39 | 0.00% | 171 | 7884 | 2.17% | 0 |
| tryb1 | ufsrat | 0 | 39 | 0.00% | 171 | 7884 | 2.17% | 0 |
| tysy | usr | 5 | 35 | 14.29% | 311 | 7194 | 4.32% | 3.3 |
| tysy | ufsrat | 10 | 35 | 28.57% | 311 | 7194 | 4.32% | 6.6 |
| urok | usr | 1 | 51 | 1.96% | 306 | 10239 | 2.99% | 0.7 |
| urok | ufsrat | 1 | 51 | 1.96% | 306 | 10239 | 2.99% | 0.7 |
| vgfr2 | usr | 3 | 129 | 2.33% | 620 | 25900 | 2.39% | 1 |
| vgfr2 | ufsrat | 0 | 129 | 0.00% | 620 | 25900 | 2.39% | 0 |
| wee1 | usr | 0 | 31 | 0.00% | 137 | 6371 | 2.15% | 0 |
| wee1 | ufsrat | 0 | 31 | 0.00% | 137 | 6371 | 2.15% | 0 |
| xiap | usr | 0 | 26 | 0.00% | 129 | 5342 | 2.41% | 0 |
| xiap | ufsrat | 0 | 26 | 0.00% | 129 | 5342 | 2.41% | 0 |
