# Supplementary material for: UFSRAT: Ultra-Fast Shape Recognition with Atom Types –The Discovery of Novel Bioactive Small Molecular Scaffolds for FKBP12 and 11βHSD1
Source: PLoS One. 2015 Feb 6;10(2):e0116570. doi: 10.1371/journal.pone.0116570 (PMC4319890; doi:10.1371/journal.pone.0116570)
Supplement: S3 Table — (DOCX) [file pone.0116570.s007.docx]

Table S3 - DUD-E profiling of USR and UFSRAT at the 1% level

| Target | Method | Hits | Possible hits | Success_rate | Library_actives | Library_total | Library_actives_proportion | Enrichment |
| --- | --- | --- | --- | --- | --- | --- | --- | --- |
| aa2ar | usr | 8 | 329 | 2.43% | 844 | 32908 | 2.56% | 0.9 |
| aa2ar | ufsrat | 35 | 329 | 10.64% | 844 | 32908 | 2.56% | 4.2 |
| abl1 | usr | 1 | 111 | 0.90% | 295 | 11180 | 2.64% | 0.3 |
| abl1 | ufsrat | 8 | 111 | 7.21% | 295 | 11180 | 2.64% | 2.7 |
| ace | usr | 4 | 179 | 2.23% | 808 | 17952 | 4.50% | 0.5 |
| ace | ufsrat | 19 | 179 | 10.61% | 808 | 17952 | 4.50% | 2.4 |
| aces | usr | 1 | 270 | 0.37% | 664 | 27037 | 2.46% | 0.2 |
| aces | ufsrat | 2 | 270 | 0.74% | 664 | 27037 | 2.46% | 0.3 |
| ada | usr | 0 | 57 | 0.00% | 262 | 5734 | 4.57% | 0 |
| ada | ufsrat | 8 | 57 | 14.04% | 262 | 5734 | 4.57% | 3.1 |
| ada17 | usr | 10 | 376 | 2.66% | 959 | 37606 | 2.55% | 1 |
| ada17 | ufsrat | 3 | 376 | 0.80% | 959 | 37606 | 2.55% | 0.3 |
| adrb1 | usr | 2 | 164 | 1.22% | 458 | 16416 | 2.79% | 0.4 |
| adrb1 | ufsrat | 0 | 164 | 0.00% | 458 | 16416 | 2.79% | 0 |
| adrb2 | usr | 2 | 157 | 1.27% | 447 | 15702 | 2.85% | 0.4 |
| adrb2 | ufsrat | 2 | 157 | 1.27% | 447 | 15702 | 2.85% | 0.4 |
| akt1 | usr | 3 | 169 | 1.78% | 423 | 16999 | 2.49% | 0.7 |
| akt1 | ufsrat | 1 | 169 | 0.59% | 423 | 16999 | 2.49% | 0.2 |
| akt2 | usr | 1 | 71 | 1.41% | 190 | 7142 | 2.66% | 0.5 |
| akt2 | ufsrat | 6 | 71 | 8.45% | 190 | 7142 | 2.66% | 3.2 |
| aldr | usr | 1 | 93 | 1.08% | 220 | 9356 | 2.35% | 0.5 |
| aldr | ufsrat | 2 | 93 | 2.15% | 220 | 9356 | 2.35% | 0.9 |
| ampc | usr | 0 | 29 | 0.00% | 62 | 2964 | 2.09% | 0 |
| ampc | ufsrat | 4 | 29 | 13.79% | 62 | 2964 | 2.09% | 6.6 |
| andr | usr | 19 | 150 | 12.67% | 523 | 15026 | 3.48% | 3.6 |
| andr | ufsrat | 26 | 150 | 17.33% | 523 | 15026 | 3.48% | 5 |
| aofb | usr | 3 | 70 | 4.29% | 168 | 7099 | 2.37% | 1.8 |
| aofb | ufsrat | 3 | 70 | 4.29% | 168 | 7099 | 2.37% | 1.8 |
| bace1 | usr | 3 | 187 | 1.60% | 485 | 18706 | 2.59% | 0.6 |
| bace1 | ufsrat | 8 | 187 | 4.28% | 485 | 18706 | 2.59% | 1.7 |
| braf | usr | 7 | 103 | 6.80% | 251 | 10349 | 2.43% | 2.8 |
| braf | ufsrat | 9 | 103 | 8.74% | 251 | 10349 | 2.43% | 3.6 |
| cah2 | usr | 7 | 325 | 2.15% | 835 | 32545 | 2.57% | 0.8 |
| cah2 | ufsrat | 14 | 325 | 4.31% | 835 | 32545 | 2.57% | 1.7 |
| casp3 | usr | 2 | 111 | 1.80% | 350 | 11172 | 3.13% | 0.6 |
| casp3 | ufsrat | 7 | 111 | 6.31% | 350 | 11172 | 3.13% | 2 |
| cdk2 | usr | 6 | 291 | 2.06% | 798 | 29126 | 2.74% | 0.8 |
| cdk2 | ufsrat | 13 | 291 | 4.47% | 798 | 29126 | 2.74% | 1.6 |
| comt | usr | 2 | 40 | 5.00% | 86 | 4012 | 2.14% | 2.3 |
| comt | ufsrat | 7 | 40 | 17.50% | 86 | 4012 | 2.14% | 8.2 |
| cp2c9 | usr | 0 | 77 | 0.00% | 183 | 7757 | 2.36% | 0 |
| cp2c9 | ufsrat | 1 | 77 | 1.30% | 183 | 7757 | 2.36% | 0.6 |
| cp3a4 | usr | 1 | 123 | 0.81% | 363 | 12303 | 2.95% | 0.3 |
| cp3a4 | ufsrat | 1 | 123 | 0.81% | 363 | 12303 | 2.95% | 0.3 |
| csf1r | usr | 11 | 127 | 8.66% | 286 | 12720 | 2.25% | 3.8 |
| csf1r | ufsrat | 5 | 127 | 3.94% | 286 | 12720 | 2.25% | 1.8 |
| cxcr4 | usr | 3 | 35 | 8.57% | 122 | 3536 | 3.45% | 2.5 |
| cxcr4 | ufsrat | 5 | 35 | 14.29% | 122 | 3536 | 3.45% | 4.1 |
| def | usr | 9 | 58 | 15.52% | 161 | 5899 | 2.73% | 5.7 |
| def | ufsrat | 7 | 58 | 12.07% | 161 | 5899 | 2.73% | 4.4 |
| dhi1 | usr | 4 | 201 | 1.99% | 519 | 20142 | 2.58% | 0.8 |
| dhi1 | ufsrat | 4 | 201 | 1.99% | 519 | 20142 | 2.58% | 0.8 |
| dpp4 | usr | 16 | 424 | 3.77% | 1079 | 42452 | 2.54% | 1.5 |
| dpp4 | ufsrat | 22 | 424 | 5.19% | 1079 | 42452 | 2.54% | 2 |
| drd3 | usr | 3 | 350 | 0.86% | 877 | 35065 | 2.50% | 0.3 |
| drd3 | ufsrat | 1 | 350 | 0.29% | 877 | 35065 | 2.50% | 0.1 |
| dyr | usr | 2 | 179 | 1.12% | 566 | 17950 | 3.15% | 0.4 |
| dyr | ufsrat | 6 | 179 | 3.35% | 566 | 17950 | 3.15% | 1.1 |
| egfr | usr | 1 | 362 | 0.28% | 832 | 36274 | 2.29% | 0.1 |
| egfr | ufsrat | 2 | 362 | 0.55% | 832 | 36274 | 2.29% | 0.2 |
| esr1 | usr | 16 | 214 | 7.48% | 627 | 21445 | 2.92% | 2.6 |
| esr1 | ufsrat | 42 | 214 | 19.63% | 627 | 21445 | 2.92% | 6.7 |
| esr2 | usr | 17 | 209 | 8.13% | 595 | 20908 | 2.85% | 2.9 |
| esr2 | ufsrat | 36 | 209 | 17.22% | 595 | 20908 | 2.85% | 6 |
| fa10 | usr | 32 | 212 | 15.09% | 792 | 21209 | 3.73% | 4 |
| fa10 | ufsrat | 13 | 212 | 6.13% | 792 | 21209 | 3.73% | 1.6 |
| fa7 | usr | 4 | 64 | 6.25% | 185 | 6487 | 2.85% | 2.2 |
| fa7 | ufsrat | 4 | 64 | 6.25% | 185 | 6487 | 2.85% | 2.2 |
| fabp4 | usr | 1 | 29 | 3.45% | 57 | 2912 | 1.96% | 1.8 |
| fabp4 | ufsrat | 1 | 29 | 3.45% | 57 | 2912 | 1.96% | 1.8 |
| fak1 | usr | 0 | 55 | 0.00% | 114 | 5516 | 2.07% | 0 |
| fak1 | ufsrat | 6 | 55 | 10.91% | 114 | 5516 | 2.07% | 5.3 |
| fkb1a | usr | 4 | 61 | 6.56% | 273 | 6105 | 4.47% | 1.5 |
| fkb1a | ufsrat | 4 | 61 | 6.56% | 273 | 6105 | 4.47% | 1.5 |
| fnta | usr | 15 | 537 | 2.79% | 1692 | 53741 | 3.15% | 0.9 |
| fnta | ufsrat | 6 | 537 | 1.12% | 1692 | 53741 | 3.15% | 0.4 |
| fpps | usr | 9 | 92 | 9.78% | 213 | 9228 | 2.31% | 4.2 |
| fpps | ufsrat | 35 | 92 | 38.04% | 213 | 9228 | 2.31% | 16.5 |
| gcr | usr | 5 | 157 | 3.18% | 563 | 15748 | 3.58% | 0.9 |
| gcr | ufsrat | 2 | 157 | 1.27% | 563 | 15748 | 3.58% | 0.4 |
| glcm | usr | 4 | 41 | 9.76% | 313 | 4150 | 7.54% | 1.3 |
| glcm | ufsrat | 6 | 41 | 14.63% | 313 | 4150 | 7.54% | 1.9 |
| gria2 | usr | 8 | 123 | 6.50% | 297 | 12358 | 2.40% | 2.7 |
| gria2 | ufsrat | 4 | 123 | 3.25% | 297 | 12358 | 2.40% | 1.4 |
| grik1 | usr | 0 | 67 | 0.00% | 152 | 6769 | 2.25% | 0 |
| grik1 | ufsrat | 0 | 67 | 0.00% | 152 | 6769 | 2.25% | 0 |
| hdac2 | usr | 0 | 106 | 0.00% | 238 | 10604 | 2.24% | 0 |
| hdac2 | ufsrat | 5 | 106 | 4.72% | 238 | 10604 | 2.24% | 2.1 |
| hdac8 | usr | 0 | 107 | 0.00% | 234 | 10748 | 2.18% | 0 |
| hdac8 | ufsrat | 4 | 107 | 3.74% | 234 | 10748 | 2.18% | 1.7 |
| hivint | usr | 0 | 69 | 0.00% | 211 | 6967 | 3.03% | 0 |
| hivint | ufsrat | 2 | 69 | 2.90% | 211 | 6967 | 3.03% | 1 |
| hivpr | usr | 33 | 376 | 8.78% | 1395 | 37673 | 3.70% | 2.4 |
| hivpr | ufsrat | 14 | 376 | 3.72% | 1395 | 37673 | 3.70% | 1 |
| hivrt | usr | 14 | 197 | 7.11% | 639 | 19773 | 3.23% | 2.2 |
| hivrt | ufsrat | 11 | 197 | 5.58% | 639 | 19773 | 3.23% | 1.7 |
| hmdh | usr | 4 | 91 | 4.40% | 299 | 9183 | 3.26% | 1.3 |
| hmdh | ufsrat | 6 | 91 | 6.59% | 299 | 9183 | 3.26% | 2 |
| hs90a | usr | 3 | 50 | 6.00% | 125 | 5067 | 2.47% | 2.4 |
| hs90a | ufsrat | 1 | 50 | 2.00% | 125 | 5067 | 2.47% | 0.8 |
| hxk4 | usr | 0 | 49 | 0.00% | 127 | 4930 | 2.58% | 0 |
| hxk4 | ufsrat | 13 | 49 | 26.53% | 127 | 4930 | 2.58% | 10.3 |
| igf1r | usr | 0 | 96 | 0.00% | 226 | 9633 | 2.35% | 0 |
| igf1r | ufsrat | 6 | 96 | 6.25% | 226 | 9633 | 2.35% | 2.7 |
| inha | usr | 0 | 23 | 0.00% | 71 | 2389 | 2.97% | 0 |
| inha | ufsrat | 4 | 23 | 17.39% | 71 | 2389 | 2.97% | 5.9 |
| ital | usr | 2 | 89 | 2.25% | 233 | 8923 | 2.61% | 0.9 |
| ital | ufsrat | 7 | 89 | 7.87% | 233 | 8923 | 2.61% | 3 |
| jak2 | usr | 7 | 67 | 10.45% | 153 | 6743 | 2.27% | 4.6 |
| jak2 | ufsrat | 4 | 67 | 5.97% | 153 | 6743 | 2.27% | 2.6 |
| kif11 | usr | 3 | 71 | 4.23% | 197 | 7109 | 2.77% | 1.5 |
| kif11 | ufsrat | 0 | 71 | 0.00% | 197 | 7109 | 2.77% | 0 |
| kit | usr | 1 | 108 | 0.93% | 252 | 10861 | 2.32% | 0.4 |
| kit | ufsrat | 0 | 108 | 0.00% | 252 | 10861 | 2.32% | 0 |
| kith | usr | 5 | 29 | 17.24% | 132 | 2998 | 4.40% | 3.9 |
| kith | ufsrat | 6 | 29 | 20.69% | 132 | 2998 | 4.40% | 4.7 |
| kpcb | usr | 13 | 90 | 14.44% | 248 | 9092 | 2.73% | 5.3 |
| kpcb | ufsrat | 8 | 90 | 8.89% | 248 | 9092 | 2.73% | 3.3 |
| lck | usr | 1 | 285 | 0.35% | 683 | 28539 | 2.39% | 0.1 |
| lck | ufsrat | 13 | 285 | 4.56% | 683 | 28539 | 2.39% | 1.9 |
| lkha4 | usr | 4 | 97 | 4.12% | 244 | 9721 | 2.51% | 1.6 |
| lkha4 | ufsrat | 2 | 97 | 2.06% | 244 | 9721 | 2.51% | 0.8 |
| mapk2 | usr | 2 | 64 | 3.13% | 206 | 6450 | 3.19% | 1 |
| mapk2 | ufsrat | 0 | 64 | 0.00% | 206 | 6450 | 3.19% | 0 |
| mcr | usr | 3 | 54 | 5.56% | 193 | 5433 | 3.55% | 1.6 |
| mcr | ufsrat | 12 | 54 | 22.22% | 193 | 5433 | 3.55% | 6.3 |
| met | usr | 16 | 116 | 13.79% | 244 | 11677 | 2.09% | 6.6 |
| met | ufsrat | 20 | 116 | 17.24% | 244 | 11677 | 2.09% | 8.2 |
| mk01 | usr | 1 | 47 | 2.13% | 139 | 4767 | 2.92% | 0.7 |
| mk01 | ufsrat | 1 | 47 | 2.13% | 139 | 4767 | 2.92% | 0.7 |
| mk10 | usr | 1 | 69 | 1.45% | 186 | 6900 | 2.70% | 0.5 |
| mk10 | ufsrat | 0 | 69 | 0.00% | 186 | 6900 | 2.70% | 0 |
| mk14 | usr | 11 | 373 | 2.95% | 915 | 37347 | 2.45% | 1.2 |
| mk14 | ufsrat | 2 | 373 | 0.54% | 915 | 37347 | 2.45% | 0.2 |
| mmp13 | usr | 16 | 390 | 4.10% | 1038 | 39046 | 2.66% | 1.5 |
| mmp13 | ufsrat | 26 | 390 | 6.67% | 1038 | 39046 | 2.66% | 2.5 |
| mp2k1 | usr | 12 | 84 | 14.29% | 242 | 8483 | 2.85% | 5 |
| mp2k1 | ufsrat | 14 | 84 | 16.67% | 242 | 8483 | 2.85% | 5.8 |
| nos1 | usr | 0 | 83 | 0.00% | 234 | 8307 | 2.82% | 0 |
| nos1 | ufsrat | 0 | 83 | 0.00% | 234 | 8307 | 2.82% | 0 |
| nram | usr | 18 | 64 | 28.13% | 222 | 6449 | 3.44% | 8.2 |
| nram | ufsrat | 26 | 64 | 40.63% | 222 | 6449 | 3.44% | 11.8 |
| pa2ga | usr | 0 | 53 | 0.00% | 127 | 5343 | 2.38% | 0 |
| pa2ga | ufsrat | 9 | 53 | 16.98% | 127 | 5343 | 2.38% | 7.1 |
| parp1 | usr | 20 | 311 | 6.43% | 742 | 31171 | 2.38% | 2.7 |
| parp1 | ufsrat | 8 | 311 | 2.57% | 742 | 31171 | 2.38% | 1.1 |
| pde5a | usr | 10 | 285 | 3.51% | 706 | 28532 | 2.47% | 1.4 |
| pde5a | ufsrat | 12 | 285 | 4.21% | 706 | 28532 | 2.47% | 1.7 |
| pgh1 | usr | 5 | 111 | 4.50% | 251 | 11193 | 2.24% | 2 |
| pgh1 | ufsrat | 3 | 111 | 2.70% | 251 | 11193 | 2.24% | 1.2 |
| pgh2 | usr | 42 | 239 | 17.57% | 531 | 23936 | 2.22% | 7.9 |
| pgh2 | ufsrat | 52 | 239 | 21.76% | 531 | 23936 | 2.22% | 9.8 |
| plk1 | usr | 2 | 70 | 2.86% | 155 | 7034 | 2.20% | 1.3 |
| plk1 | ufsrat | 1 | 70 | 1.43% | 155 | 7034 | 2.20% | 0.7 |
| pnph | usr | 7 | 72 | 9.72% | 233 | 7249 | 3.21% | 3 |
| pnph | ufsrat | 11 | 72 | 15.28% | 233 | 7249 | 3.21% | 4.8 |
| ppara | usr | 30 | 203 | 14.78% | 544 | 20375 | 2.67% | 5.5 |
| ppara | ufsrat | 15 | 203 | 7.39% | 544 | 20375 | 2.67% | 2.8 |
| ppard | usr | 0 | 135 | 0.00% | 288 | 13520 | 2.13% | 0 |
| ppard | ufsrat | 2 | 135 | 1.48% | 288 | 13520 | 2.13% | 0.7 |
| pparg | usr | 9 | 265 | 3.40% | 723 | 26590 | 2.72% | 1.3 |
| pparg | ufsrat | 4 | 265 | 1.51% | 723 | 26590 | 2.72% | 0.6 |
| prgr | usr | 21 | 162 | 12.96% | 444 | 16258 | 2.73% | 4.7 |
| prgr | ufsrat | 21 | 162 | 12.96% | 444 | 16258 | 2.73% | 4.7 |
| ptn1 | usr | 1 | 76 | 1.32% | 225 | 7658 | 2.94% | 0.4 |
| ptn1 | ufsrat | 2 | 76 | 2.63% | 225 | 7658 | 2.94% | 0.9 |
| pur2 | usr | 0 | 29 | 0.00% | 201 | 2926 | 6.87% | 0 |
| pur2 | ufsrat | 0 | 29 | 0.00% | 201 | 2926 | 6.87% | 0 |
| pygm | usr | 2 | 41 | 4.88% | 114 | 4159 | 2.74% | 1.8 |
| pygm | ufsrat | 11 | 41 | 26.83% | 114 | 4159 | 2.74% | 9.8 |
| pyrd | usr | 12 | 67 | 17.91% | 134 | 6782 | 1.98% | 9 |
| pyrd | ufsrat | 26 | 67 | 38.81% | 134 | 6782 | 1.98% | 19.6 |
| reni | usr | 3 | 73 | 4.11% | 387 | 7371 | 5.25% | 0.8 |
| reni | ufsrat | 4 | 73 | 5.48% | 387 | 7371 | 5.25% | 1 |
| rock1 | usr | 0 | 65 | 0.00% | 203 | 6580 | 3.09% | 0 |
| rock1 | ufsrat | 4 | 65 | 6.15% | 203 | 6580 | 3.09% | 2 |
| rxra | usr | 1 | 78 | 1.28% | 162 | 7869 | 2.06% | 0.6 |
| rxra | ufsrat | 3 | 78 | 3.85% | 162 | 7869 | 2.06% | 1.9 |
| sahh | usr | 3 | 36 | 8.33% | 190 | 3673 | 5.17% | 1.6 |
| sahh | ufsrat | 8 | 36 | 22.22% | 190 | 3673 | 5.17% | 4.3 |
| src | usr | 3 | 357 | 0.84% | 831 | 35790 | 2.32% | 0.4 |
| src | ufsrat | 28 | 357 | 7.84% | 831 | 35790 | 2.32% | 3.4 |
| tgfr1 | usr | 9 | 89 | 10.11% | 281 | 8958 | 3.14% | 3.2 |
| tgfr1 | ufsrat | 18 | 89 | 20.22% | 281 | 8958 | 3.14% | 6.4 |
| thb | usr | 1 | 78 | 1.28% | 168 | 7821 | 2.15% | 0.6 |
| thb | ufsrat | 12 | 78 | 15.38% | 168 | 7821 | 2.15% | 7.2 |
| thrb | usr | 5 | 281 | 1.78% | 861 | 28182 | 3.06% | 0.6 |
| thrb | ufsrat | 4 | 281 | 1.42% | 861 | 28182 | 3.06% | 0.5 |
| try1 | usr | 13 | 269 | 4.83% | 758 | 26977 | 2.81% | 1.7 |
| try1 | ufsrat | 4 | 269 | 1.49% | 758 | 26977 | 2.81% | 0.5 |
| tryb1 | usr | 0 | 78 | 0.00% | 171 | 7884 | 2.17% | 0 |
| tryb1 | ufsrat | 0 | 78 | 0.00% | 171 | 7884 | 2.17% | 0 |
| tysy | usr | 12 | 71 | 16.90% | 311 | 7194 | 4.32% | 3.9 |
| tysy | ufsrat | 11 | 71 | 15.49% | 311 | 7194 | 4.32% | 3.6 |
| urok | usr | 4 | 102 | 3.92% | 306 | 10239 | 2.99% | 1.3 |
| urok | ufsrat | 1 | 102 | 0.98% | 306 | 10239 | 2.99% | 0.3 |
| vgfr2 | usr | 8 | 259 | 3.09% | 620 | 25900 | 2.39% | 1.3 |
| vgfr2 | ufsrat | 1 | 259 | 0.39% | 620 | 25900 | 2.39% | 0.2 |
| wee1 | usr | 0 | 63 | 0.00% | 137 | 6371 | 2.15% | 0 |
| wee1 | ufsrat | 0 | 63 | 0.00% | 137 | 6371 | 2.15% | 0 |
| xiap | usr | 3 | 53 | 5.66% | 129 | 5342 | 2.41% | 2.3 |
| xiap | ufsrat | 1 | 53 | 1.89% | 129 | 5342 | 2.41% | 0.8 |
