# Supplementary material for: UFSRAT: Ultra-Fast Shape Recognition with Atom Types –The Discovery of Novel Bioactive Small Molecular Scaffolds for FKBP12 and 11βHSD1
Source: PLoS One. 2015 Feb 6;10(2):e0116570. doi: 10.1371/journal.pone.0116570 (PMC4319890; doi:10.1371/journal.pone.0116570)
Supplement: S4 Table — (DOCX) [file pone.0116570.s008.docx]

Table S4 - DUD-E profiling of USR and UFSRAT at the 2% level

| Target | Method | Hits | Possible hits | Success_rate | Library_actives | Library_total | Library_actives_proportion | Enrichment |
| --- | --- | --- | --- | --- | --- | --- | --- | --- |
| aa2ar | usr | 13 | 658 | 1.98% | 844 | 32908 | 2.56% | 0.8 |
| aa2ar | ufsrat | 50 | 658 | 7.60% | 844 | 32908 | 2.56% | 3 |
| abl1 | usr | 4 | 222 | 1.80% | 295 | 11180 | 2.64% | 0.7 |
| abl1 | ufsrat | 14 | 222 | 6.31% | 295 | 11180 | 2.64% | 2.4 |
| ace | usr | 11 | 358 | 3.07% | 808 | 17952 | 4.50% | 0.7 |
| ace | ufsrat | 29 | 358 | 8.10% | 808 | 17952 | 4.50% | 1.8 |
| aces | usr | 2 | 540 | 0.37% | 664 | 27037 | 2.46% | 0.2 |
| aces | ufsrat | 5 | 540 | 0.93% | 664 | 27037 | 2.46% | 0.4 |
| ada | usr | 2 | 114 | 1.75% | 262 | 5734 | 4.57% | 0.4 |
| ada | ufsrat | 11 | 114 | 9.65% | 262 | 5734 | 4.57% | 2.1 |
| ada17 | usr | 18 | 752 | 2.39% | 959 | 37606 | 2.55% | 0.9 |
| ada17 | ufsrat | 9 | 752 | 1.20% | 959 | 37606 | 2.55% | 0.5 |
| adrb1 | usr | 2 | 328 | 0.61% | 458 | 16416 | 2.79% | 0.2 |
| adrb1 | ufsrat | 0 | 328 | 0.00% | 458 | 16416 | 2.79% | 0 |
| adrb2 | usr | 4 | 314 | 1.27% | 447 | 15702 | 2.85% | 0.4 |
| adrb2 | ufsrat | 2 | 314 | 0.64% | 447 | 15702 | 2.85% | 0.2 |
| akt1 | usr | 7 | 338 | 2.07% | 423 | 16999 | 2.49% | 0.8 |
| akt1 | ufsrat | 1 | 338 | 0.30% | 423 | 16999 | 2.49% | 0.1 |
| akt2 | usr | 1 | 142 | 0.70% | 190 | 7142 | 2.66% | 0.3 |
| akt2 | ufsrat | 7 | 142 | 4.93% | 190 | 7142 | 2.66% | 1.9 |
| aldr | usr | 2 | 186 | 1.08% | 220 | 9356 | 2.35% | 0.5 |
| aldr | ufsrat | 6 | 186 | 3.23% | 220 | 9356 | 2.35% | 1.4 |
| ampc | usr | 0 | 58 | 0.00% | 62 | 2964 | 2.09% | 0 |
| ampc | ufsrat | 6 | 58 | 10.34% | 62 | 2964 | 2.09% | 4.9 |
| andr | usr | 35 | 300 | 11.67% | 523 | 15026 | 3.48% | 3.4 |
| andr | ufsrat | 43 | 300 | 14.33% | 523 | 15026 | 3.48% | 4.1 |
| aofb | usr | 4 | 140 | 2.86% | 168 | 7099 | 2.37% | 1.2 |
| aofb | ufsrat | 3 | 140 | 2.14% | 168 | 7099 | 2.37% | 0.9 |
| bace1 | usr | 7 | 374 | 1.87% | 485 | 18706 | 2.59% | 0.7 |
| bace1 | ufsrat | 18 | 374 | 4.81% | 485 | 18706 | 2.59% | 1.9 |
| braf | usr | 12 | 206 | 5.83% | 251 | 10349 | 2.43% | 2.4 |
| braf | ufsrat | 17 | 206 | 8.25% | 251 | 10349 | 2.43% | 3.4 |
| cah2 | usr | 9 | 650 | 1.38% | 835 | 32545 | 2.57% | 0.5 |
| cah2 | ufsrat | 19 | 650 | 2.92% | 835 | 32545 | 2.57% | 1.1 |
| casp3 | usr | 4 | 222 | 1.80% | 350 | 11172 | 3.13% | 0.6 |
| casp3 | ufsrat | 12 | 222 | 5.41% | 350 | 11172 | 3.13% | 1.7 |
| cdk2 | usr | 8 | 582 | 1.37% | 798 | 29126 | 2.74% | 0.5 |
| cdk2 | ufsrat | 24 | 582 | 4.12% | 798 | 29126 | 2.74% | 1.5 |
| comt | usr | 3 | 80 | 3.75% | 86 | 4012 | 2.14% | 1.8 |
| comt | ufsrat | 9 | 80 | 11.25% | 86 | 4012 | 2.14% | 5.3 |
| cp2c9 | usr | 0 | 154 | 0.00% | 183 | 7757 | 2.36% | 0 |
| cp2c9 | ufsrat | 2 | 154 | 1.30% | 183 | 7757 | 2.36% | 0.6 |
| cp3a4 | usr | 6 | 246 | 2.44% | 363 | 12303 | 2.95% | 0.8 |
| cp3a4 | ufsrat | 1 | 246 | 0.41% | 363 | 12303 | 2.95% | 0.1 |
| csf1r | usr | 18 | 254 | 7.09% | 286 | 12720 | 2.25% | 3.2 |
| csf1r | ufsrat | 20 | 254 | 7.87% | 286 | 12720 | 2.25% | 3.5 |
| cxcr4 | usr | 7 | 70 | 10.00% | 122 | 3536 | 3.45% | 2.9 |
| cxcr4 | ufsrat | 14 | 70 | 20.00% | 122 | 3536 | 3.45% | 5.8 |
| def | usr | 14 | 116 | 12.07% | 161 | 5899 | 2.73% | 4.4 |
| def | ufsrat | 13 | 116 | 11.21% | 161 | 5899 | 2.73% | 4.1 |
| dhi1 | usr | 7 | 402 | 1.74% | 519 | 20142 | 2.58% | 0.7 |
| dhi1 | ufsrat | 12 | 402 | 2.99% | 519 | 20142 | 2.58% | 1.2 |
| dpp4 | usr | 31 | 848 | 3.66% | 1079 | 42452 | 2.54% | 1.4 |
| dpp4 | ufsrat | 44 | 848 | 5.19% | 1079 | 42452 | 2.54% | 2 |
| drd3 | usr | 5 | 700 | 0.71% | 877 | 35065 | 2.50% | 0.3 |
| drd3 | ufsrat | 2 | 700 | 0.29% | 877 | 35065 | 2.50% | 0.1 |
| dyr | usr | 5 | 358 | 1.40% | 566 | 17950 | 3.15% | 0.4 |
| dyr | ufsrat | 7 | 358 | 1.96% | 566 | 17950 | 3.15% | 0.6 |
| egfr | usr | 6 | 724 | 0.83% | 832 | 36274 | 2.29% | 0.4 |
| egfr | ufsrat | 11 | 724 | 1.52% | 832 | 36274 | 2.29% | 0.7 |
| esr1 | usr | 22 | 428 | 5.14% | 627 | 21445 | 2.92% | 1.8 |
| esr1 | ufsrat | 65 | 428 | 15.19% | 627 | 21445 | 2.92% | 5.2 |
| esr2 | usr | 30 | 418 | 7.18% | 595 | 20908 | 2.85% | 2.5 |
| esr2 | ufsrat | 75 | 418 | 17.94% | 595 | 20908 | 2.85% | 6.3 |
| fa10 | usr | 57 | 424 | 13.44% | 792 | 21209 | 3.73% | 3.6 |
| fa10 | ufsrat | 23 | 424 | 5.42% | 792 | 21209 | 3.73% | 1.5 |
| fa7 | usr | 4 | 128 | 3.13% | 185 | 6487 | 2.85% | 1.1 |
| fa7 | ufsrat | 7 | 128 | 5.47% | 185 | 6487 | 2.85% | 1.9 |
| fabp4 | usr | 1 | 58 | 1.72% | 57 | 2912 | 1.96% | 0.9 |
| fabp4 | ufsrat | 1 | 58 | 1.72% | 57 | 2912 | 1.96% | 0.9 |
| fak1 | usr | 1 | 110 | 0.91% | 114 | 5516 | 2.07% | 0.4 |
| fak1 | ufsrat | 12 | 110 | 10.91% | 114 | 5516 | 2.07% | 5.3 |
| fkb1a | usr | 7 | 122 | 5.74% | 273 | 6105 | 4.47% | 1.3 |
| fkb1a | ufsrat | 11 | 122 | 9.02% | 273 | 6105 | 4.47% | 2 |
| fnta | usr | 34 | 1074 | 3.17% | 1692 | 53741 | 3.15% | 1 |
| fnta | ufsrat | 15 | 1074 | 1.40% | 1692 | 53741 | 3.15% | 0.4 |
| fpps | usr | 16 | 184 | 8.70% | 213 | 9228 | 2.31% | 3.8 |
| fpps | ufsrat | 52 | 184 | 28.26% | 213 | 9228 | 2.31% | 12.2 |
| gcr | usr | 8 | 314 | 2.55% | 563 | 15748 | 3.58% | 0.7 |
| gcr | ufsrat | 5 | 314 | 1.59% | 563 | 15748 | 3.58% | 0.4 |
| glcm | usr | 4 | 82 | 4.88% | 313 | 4150 | 7.54% | 0.6 |
| glcm | ufsrat | 7 | 82 | 8.54% | 313 | 4150 | 7.54% | 1.1 |
| gria2 | usr | 10 | 246 | 4.07% | 297 | 12358 | 2.40% | 1.7 |
| gria2 | ufsrat | 5 | 246 | 2.03% | 297 | 12358 | 2.40% | 0.8 |
| grik1 | usr | 0 | 134 | 0.00% | 152 | 6769 | 2.25% | 0 |
| grik1 | ufsrat | 0 | 134 | 0.00% | 152 | 6769 | 2.25% | 0 |
| hdac2 | usr | 0 | 212 | 0.00% | 238 | 10604 | 2.24% | 0 |
| hdac2 | ufsrat | 5 | 212 | 2.36% | 238 | 10604 | 2.24% | 1.1 |
| hdac8 | usr | 0 | 214 | 0.00% | 234 | 10748 | 2.18% | 0 |
| hdac8 | ufsrat | 4 | 214 | 1.87% | 234 | 10748 | 2.18% | 0.9 |
| hivint | usr | 0 | 138 | 0.00% | 211 | 6967 | 3.03% | 0 |
| hivint | ufsrat | 6 | 138 | 4.35% | 211 | 6967 | 3.03% | 1.4 |
| hivpr | usr | 54 | 752 | 7.18% | 1395 | 37673 | 3.70% | 1.9 |
| hivpr | ufsrat | 35 | 752 | 4.65% | 1395 | 37673 | 3.70% | 1.3 |
| hivrt | usr | 25 | 394 | 6.35% | 639 | 19773 | 3.23% | 2 |
| hivrt | ufsrat | 20 | 394 | 5.08% | 639 | 19773 | 3.23% | 1.6 |
| hmdh | usr | 13 | 182 | 7.14% | 299 | 9183 | 3.26% | 2.2 |
| hmdh | ufsrat | 7 | 182 | 3.85% | 299 | 9183 | 3.26% | 1.2 |
| hs90a | usr | 5 | 100 | 5.00% | 125 | 5067 | 2.47% | 2 |
| hs90a | ufsrat | 3 | 100 | 3.00% | 125 | 5067 | 2.47% | 1.2 |
| hxk4 | usr | 0 | 98 | 0.00% | 127 | 4930 | 2.58% | 0 |
| hxk4 | ufsrat | 19 | 98 | 19.39% | 127 | 4930 | 2.58% | 7.5 |
| igf1r | usr | 0 | 192 | 0.00% | 226 | 9633 | 2.35% | 0 |
| igf1r | ufsrat | 7 | 192 | 3.65% | 226 | 9633 | 2.35% | 1.6 |
| inha | usr | 0 | 46 | 0.00% | 71 | 2389 | 2.97% | 0 |
| inha | ufsrat | 7 | 46 | 15.22% | 71 | 2389 | 2.97% | 5.1 |
| ital | usr | 6 | 178 | 3.37% | 233 | 8923 | 2.61% | 1.3 |
| ital | ufsrat | 9 | 178 | 5.06% | 233 | 8923 | 2.61% | 1.9 |
| jak2 | usr | 7 | 134 | 5.22% | 153 | 6743 | 2.27% | 2.3 |
| jak2 | ufsrat | 6 | 134 | 4.48% | 153 | 6743 | 2.27% | 2 |
| kif11 | usr | 3 | 142 | 2.11% | 197 | 7109 | 2.77% | 0.8 |
| kif11 | ufsrat | 1 | 142 | 0.70% | 197 | 7109 | 2.77% | 0.3 |
| kit | usr | 1 | 216 | 0.46% | 252 | 10861 | 2.32% | 0.2 |
| kit | ufsrat | 0 | 216 | 0.00% | 252 | 10861 | 2.32% | 0 |
| kith | usr | 7 | 58 | 12.07% | 132 | 2998 | 4.40% | 2.7 |
| kith | ufsrat | 7 | 58 | 12.07% | 132 | 2998 | 4.40% | 2.7 |
| kpcb | usr | 18 | 180 | 10.00% | 248 | 9092 | 2.73% | 3.7 |
| kpcb | ufsrat | 14 | 180 | 7.78% | 248 | 9092 | 2.73% | 2.8 |
| lck | usr | 3 | 570 | 0.53% | 683 | 28539 | 2.39% | 0.2 |
| lck | ufsrat | 19 | 570 | 3.33% | 683 | 28539 | 2.39% | 1.4 |
| lkha4 | usr | 9 | 194 | 4.64% | 244 | 9721 | 2.51% | 1.8 |
| lkha4 | ufsrat | 2 | 194 | 1.03% | 244 | 9721 | 2.51% | 0.4 |
| mapk2 | usr | 3 | 128 | 2.34% | 206 | 6450 | 3.19% | 0.7 |
| mapk2 | ufsrat | 6 | 128 | 4.69% | 206 | 6450 | 3.19% | 1.5 |
| mcr | usr | 8 | 108 | 7.41% | 193 | 5433 | 3.55% | 2.1 |
| mcr | ufsrat | 14 | 108 | 12.96% | 193 | 5433 | 3.55% | 3.7 |
| met | usr | 29 | 232 | 12.50% | 244 | 11677 | 2.09% | 6 |
| met | ufsrat | 31 | 232 | 13.36% | 244 | 11677 | 2.09% | 6.4 |
| mk01 | usr | 3 | 94 | 3.19% | 139 | 4767 | 2.92% | 1.1 |
| mk01 | ufsrat | 5 | 94 | 5.32% | 139 | 4767 | 2.92% | 1.8 |
| mk10 | usr | 1 | 138 | 0.72% | 186 | 6900 | 2.70% | 0.3 |
| mk10 | ufsrat | 0 | 138 | 0.00% | 186 | 6900 | 2.70% | 0 |
| mk14 | usr | 26 | 746 | 3.49% | 915 | 37347 | 2.45% | 1.4 |
| mk14 | ufsrat | 5 | 746 | 0.67% | 915 | 37347 | 2.45% | 0.3 |
| mmp13 | usr | 30 | 780 | 3.85% | 1038 | 39046 | 2.66% | 1.4 |
| mmp13 | ufsrat | 56 | 780 | 7.18% | 1038 | 39046 | 2.66% | 2.7 |
| mp2k1 | usr | 15 | 168 | 8.93% | 242 | 8483 | 2.85% | 3.1 |
| mp2k1 | ufsrat | 18 | 168 | 10.71% | 242 | 8483 | 2.85% | 3.8 |
| nos1 | usr | 0 | 166 | 0.00% | 234 | 8307 | 2.82% | 0 |
| nos1 | ufsrat | 1 | 166 | 0.60% | 234 | 8307 | 2.82% | 0.2 |
| nram | usr | 26 | 128 | 20.31% | 222 | 6449 | 3.44% | 5.9 |
| nram | ufsrat | 35 | 128 | 27.34% | 222 | 6449 | 3.44% | 7.9 |
| pa2ga | usr | 1 | 106 | 0.94% | 127 | 5343 | 2.38% | 0.4 |
| pa2ga | ufsrat | 16 | 106 | 15.09% | 127 | 5343 | 2.38% | 6.3 |
| parp1 | usr | 28 | 622 | 4.50% | 742 | 31171 | 2.38% | 1.9 |
| parp1 | ufsrat | 19 | 622 | 3.05% | 742 | 31171 | 2.38% | 1.3 |
| pde5a | usr | 14 | 570 | 2.46% | 706 | 28532 | 2.47% | 1 |
| pde5a | ufsrat | 22 | 570 | 3.86% | 706 | 28532 | 2.47% | 1.6 |
| pgh1 | usr | 9 | 222 | 4.05% | 251 | 11193 | 2.24% | 1.8 |
| pgh1 | ufsrat | 7 | 222 | 3.15% | 251 | 11193 | 2.24% | 1.4 |
| pgh2 | usr | 62 | 478 | 12.97% | 531 | 23936 | 2.22% | 5.8 |
| pgh2 | ufsrat | 57 | 478 | 11.92% | 531 | 23936 | 2.22% | 5.4 |
| plk1 | usr | 4 | 140 | 2.86% | 155 | 7034 | 2.20% | 1.3 |
| plk1 | ufsrat | 1 | 140 | 0.71% | 155 | 7034 | 2.20% | 0.3 |
| pnph | usr | 12 | 144 | 8.33% | 233 | 7249 | 3.21% | 2.6 |
| pnph | ufsrat | 12 | 144 | 8.33% | 233 | 7249 | 3.21% | 2.6 |
| ppara | usr | 48 | 406 | 11.82% | 544 | 20375 | 2.67% | 4.4 |
| ppara | ufsrat | 18 | 406 | 4.43% | 544 | 20375 | 2.67% | 1.7 |
| ppard | usr | 0 | 270 | 0.00% | 288 | 13520 | 2.13% | 0 |
| ppard | ufsrat | 5 | 270 | 1.85% | 288 | 13520 | 2.13% | 0.9 |
| pparg | usr | 10 | 530 | 1.89% | 723 | 26590 | 2.72% | 0.7 |
| pparg | ufsrat | 10 | 530 | 1.89% | 723 | 26590 | 2.72% | 0.7 |
| prgr | usr | 34 | 324 | 10.49% | 444 | 16258 | 2.73% | 3.8 |
| prgr | ufsrat | 32 | 324 | 9.88% | 444 | 16258 | 2.73% | 3.6 |
| ptn1 | usr | 1 | 152 | 0.66% | 225 | 7658 | 2.94% | 0.2 |
| ptn1 | ufsrat | 3 | 152 | 1.97% | 225 | 7658 | 2.94% | 0.7 |
| pur2 | usr | 0 | 58 | 0.00% | 201 | 2926 | 6.87% | 0 |
| pur2 | ufsrat | 0 | 58 | 0.00% | 201 | 2926 | 6.87% | 0 |
| pygm | usr | 2 | 82 | 2.44% | 114 | 4159 | 2.74% | 0.9 |
| pygm | ufsrat | 15 | 82 | 18.29% | 114 | 4159 | 2.74% | 6.7 |
| pyrd | usr | 20 | 134 | 14.93% | 134 | 6782 | 1.98% | 7.5 |
| pyrd | ufsrat | 30 | 134 | 22.39% | 134 | 6782 | 1.98% | 11.3 |
| reni | usr | 5 | 146 | 3.42% | 387 | 7371 | 5.25% | 0.7 |
| reni | ufsrat | 7 | 146 | 4.79% | 387 | 7371 | 5.25% | 0.9 |
| rock1 | usr | 3 | 130 | 2.31% | 203 | 6580 | 3.09% | 0.7 |
| rock1 | ufsrat | 7 | 130 | 5.38% | 203 | 6580 | 3.09% | 1.7 |
| rxra | usr | 2 | 156 | 1.28% | 162 | 7869 | 2.06% | 0.6 |
| rxra | ufsrat | 7 | 156 | 4.49% | 162 | 7869 | 2.06% | 2.2 |
| sahh | usr | 8 | 72 | 11.11% | 190 | 3673 | 5.17% | 2.1 |
| sahh | ufsrat | 12 | 72 | 16.67% | 190 | 3673 | 5.17% | 3.2 |
| src | usr | 6 | 714 | 0.84% | 831 | 35790 | 2.32% | 0.4 |
| src | ufsrat | 41 | 714 | 5.74% | 831 | 35790 | 2.32% | 2.5 |
| tgfr1 | usr | 11 | 178 | 6.18% | 281 | 8958 | 3.14% | 2 |
| tgfr1 | ufsrat | 25 | 178 | 14.04% | 281 | 8958 | 3.14% | 4.5 |
| thb | usr | 2 | 156 | 1.28% | 168 | 7821 | 2.15% | 0.6 |
| thb | ufsrat | 19 | 156 | 12.18% | 168 | 7821 | 2.15% | 5.7 |
| thrb | usr | 8 | 562 | 1.42% | 861 | 28182 | 3.06% | 0.5 |
| thrb | ufsrat | 7 | 562 | 1.25% | 861 | 28182 | 3.06% | 0.4 |
| try1 | usr | 25 | 538 | 4.65% | 758 | 26977 | 2.81% | 1.7 |
| try1 | ufsrat | 6 | 538 | 1.12% | 758 | 26977 | 2.81% | 0.4 |
| tryb1 | usr | 1 | 156 | 0.64% | 171 | 7884 | 2.17% | 0.3 |
| tryb1 | ufsrat | 0 | 156 | 0.00% | 171 | 7884 | 2.17% | 0 |
| tysy | usr | 19 | 142 | 13.38% | 311 | 7194 | 4.32% | 3.1 |
| tysy | ufsrat | 15 | 142 | 10.56% | 311 | 7194 | 4.32% | 2.4 |
| urok | usr | 5 | 204 | 2.45% | 306 | 10239 | 2.99% | 0.8 |
| urok | ufsrat | 12 | 204 | 5.88% | 306 | 10239 | 2.99% | 2 |
| vgfr2 | usr | 15 | 518 | 2.90% | 620 | 25900 | 2.39% | 1.2 |
| vgfr2 | ufsrat | 1 | 518 | 0.19% | 620 | 25900 | 2.39% | 0.1 |
| wee1 | usr | 0 | 126 | 0.00% | 137 | 6371 | 2.15% | 0 |
| wee1 | ufsrat | 1 | 126 | 0.79% | 137 | 6371 | 2.15% | 0.4 |
| xiap | usr | 4 | 106 | 3.77% | 129 | 5342 | 2.41% | 1.6 |
| xiap | ufsrat | 2 | 106 | 1.89% | 129 | 5342 | 2.41% | 0.8 |
