# Supplementary material for: UFSRAT: Ultra-Fast Shape Recognition with Atom Types –The Discovery of Novel Bioactive Small Molecular Scaffolds for FKBP12 and 11βHSD1
Source: PLoS One. 2015 Feb 6;10(2):e0116570. doi: 10.1371/journal.pone.0116570 (PMC4319890; doi:10.1371/journal.pone.0116570)
Supplement: S5 Table — (DOCX) [file pone.0116570.s009.docx]

Table S5 - DUD-E profiling of USR and UFSRAT at the 5% level

| Target | Method | Hits | Possible hits | Success_rate | Library_actives | Library_total | Library_actives_proportion | Enrichment |
| --- | --- | --- | --- | --- | --- | --- | --- | --- |
| aa2ar | usr | 28 | 1645 | 1.70% | 844 | 32908 | 2.56% | 0.7 |
| aa2ar | ufsrat | 89 | 1645 | 5.41% | 844 | 32908 | 2.56% | 2.1 |
| abl1 | usr | 10 | 555 | 1.80% | 295 | 11180 | 2.64% | 0.7 |
| abl1 | ufsrat | 24 | 555 | 4.32% | 295 | 11180 | 2.64% | 1.6 |
| ace | usr | 20 | 895 | 2.23% | 808 | 17952 | 4.50% | 0.5 |
| ace | ufsrat | 57 | 895 | 6.37% | 808 | 17952 | 4.50% | 1.4 |
| aces | usr | 7 | 1350 | 0.52% | 664 | 27037 | 2.46% | 0.2 |
| aces | ufsrat | 19 | 1350 | 1.41% | 664 | 27037 | 2.46% | 0.6 |
| ada | usr | 5 | 285 | 1.75% | 262 | 5734 | 4.57% | 0.4 |
| ada | ufsrat | 16 | 285 | 5.61% | 262 | 5734 | 4.57% | 1.2 |
| ada17 | usr | 34 | 1880 | 1.81% | 959 | 37606 | 2.55% | 0.7 |
| ada17 | ufsrat | 24 | 1880 | 1.28% | 959 | 37606 | 2.55% | 0.5 |
| adrb1 | usr | 3 | 820 | 0.37% | 458 | 16416 | 2.79% | 0.1 |
| adrb1 | ufsrat | 1 | 820 | 0.12% | 458 | 16416 | 2.79% | 0 |
| adrb2 | usr | 5 | 785 | 0.64% | 447 | 15702 | 2.85% | 0.2 |
| adrb2 | ufsrat | 4 | 785 | 0.51% | 447 | 15702 | 2.85% | 0.2 |
| akt1 | usr | 10 | 845 | 1.18% | 423 | 16999 | 2.49% | 0.5 |
| akt1 | ufsrat | 6 | 845 | 0.71% | 423 | 16999 | 2.49% | 0.3 |
| akt2 | usr | 5 | 355 | 1.41% | 190 | 7142 | 2.66% | 0.5 |
| akt2 | ufsrat | 7 | 355 | 1.97% | 190 | 7142 | 2.66% | 0.7 |
| aldr | usr | 4 | 465 | 0.86% | 220 | 9356 | 2.35% | 0.4 |
| aldr | ufsrat | 16 | 465 | 3.44% | 220 | 9356 | 2.35% | 1.5 |
| ampc | usr | 2 | 145 | 1.38% | 62 | 2964 | 2.09% | 0.7 |
| ampc | ufsrat | 11 | 145 | 7.59% | 62 | 2964 | 2.09% | 3.6 |
| andr | usr | 52 | 750 | 6.93% | 523 | 15026 | 3.48% | 2 |
| andr | ufsrat | 67 | 750 | 8.93% | 523 | 15026 | 3.48% | 2.6 |
| aofb | usr | 7 | 350 | 2.00% | 168 | 7099 | 2.37% | 0.8 |
| aofb | ufsrat | 3 | 350 | 0.86% | 168 | 7099 | 2.37% | 0.4 |
| bace1 | usr | 17 | 935 | 1.82% | 485 | 18706 | 2.59% | 0.7 |
| bace1 | ufsrat | 39 | 935 | 4.17% | 485 | 18706 | 2.59% | 1.6 |
| braf | usr | 14 | 515 | 2.72% | 251 | 10349 | 2.43% | 1.1 |
| braf | ufsrat | 27 | 515 | 5.24% | 251 | 10349 | 2.43% | 2.2 |
| cah2 | usr | 20 | 1625 | 1.23% | 835 | 32545 | 2.57% | 0.5 |
| cah2 | ufsrat | 35 | 1625 | 2.15% | 835 | 32545 | 2.57% | 0.8 |
| casp3 | usr | 12 | 555 | 2.16% | 350 | 11172 | 3.13% | 0.7 |
| casp3 | ufsrat | 29 | 555 | 5.23% | 350 | 11172 | 3.13% | 1.7 |
| cdk2 | usr | 27 | 1455 | 1.86% | 798 | 29126 | 2.74% | 0.7 |
| cdk2 | ufsrat | 43 | 1455 | 2.96% | 798 | 29126 | 2.74% | 1.1 |
| comt | usr | 5 | 200 | 2.50% | 86 | 4012 | 2.14% | 1.2 |
| comt | ufsrat | 11 | 200 | 5.50% | 86 | 4012 | 2.14% | 2.6 |
| cp2c9 | usr | 2 | 385 | 0.52% | 183 | 7757 | 2.36% | 0.2 |
| cp2c9 | ufsrat | 7 | 385 | 1.82% | 183 | 7757 | 2.36% | 0.8 |
| cp3a4 | usr | 14 | 615 | 2.28% | 363 | 12303 | 2.95% | 0.8 |
| cp3a4 | ufsrat | 9 | 615 | 1.46% | 363 | 12303 | 2.95% | 0.5 |
| csf1r | usr | 26 | 635 | 4.09% | 286 | 12720 | 2.25% | 1.8 |
| csf1r | ufsrat | 31 | 635 | 4.88% | 286 | 12720 | 2.25% | 2.2 |
| cxcr4 | usr | 11 | 175 | 6.29% | 122 | 3536 | 3.45% | 1.8 |
| cxcr4 | ufsrat | 25 | 175 | 14.29% | 122 | 3536 | 3.45% | 4.1 |
| def | usr | 23 | 290 | 7.93% | 161 | 5899 | 2.73% | 2.9 |
| def | ufsrat | 27 | 290 | 9.31% | 161 | 5899 | 2.73% | 3.4 |
| dhi1 | usr | 19 | 1005 | 1.89% | 519 | 20142 | 2.58% | 0.7 |
| dhi1 | ufsrat | 37 | 1005 | 3.68% | 519 | 20142 | 2.58% | 1.4 |
| dpp4 | usr | 57 | 2120 | 2.69% | 1079 | 42452 | 2.54% | 1.1 |
| dpp4 | ufsrat | 100 | 2120 | 4.72% | 1079 | 42452 | 2.54% | 1.9 |
| drd3 | usr | 15 | 1750 | 0.86% | 877 | 35065 | 2.50% | 0.3 |
| drd3 | ufsrat | 5 | 1750 | 0.29% | 877 | 35065 | 2.50% | 0.1 |
| dyr | usr | 13 | 895 | 1.45% | 566 | 17950 | 3.15% | 0.5 |
| dyr | ufsrat | 12 | 895 | 1.34% | 566 | 17950 | 3.15% | 0.4 |
| egfr | usr | 16 | 1810 | 0.88% | 832 | 36274 | 2.29% | 0.4 |
| egfr | ufsrat | 38 | 1810 | 2.10% | 832 | 36274 | 2.29% | 0.9 |
| esr1 | usr | 42 | 1070 | 3.93% | 627 | 21445 | 2.92% | 1.3 |
| esr1 | ufsrat | 110 | 1070 | 10.28% | 627 | 21445 | 2.92% | 3.5 |
| esr2 | usr | 48 | 1045 | 4.59% | 595 | 20908 | 2.85% | 1.6 |
| esr2 | ufsrat | 164 | 1045 | 15.69% | 595 | 20908 | 2.85% | 5.5 |
| fa10 | usr | 105 | 1060 | 9.91% | 792 | 21209 | 3.73% | 2.7 |
| fa10 | ufsrat | 53 | 1060 | 5.00% | 792 | 21209 | 3.73% | 1.3 |
| fa7 | usr | 10 | 320 | 3.13% | 185 | 6487 | 2.85% | 1.1 |
| fa7 | ufsrat | 13 | 320 | 4.06% | 185 | 6487 | 2.85% | 1.4 |
| fabp4 | usr | 2 | 145 | 1.38% | 57 | 2912 | 1.96% | 0.7 |
| fabp4 | ufsrat | 11 | 145 | 7.59% | 57 | 2912 | 1.96% | 3.9 |
| fak1 | usr | 9 | 275 | 3.27% | 114 | 5516 | 2.07% | 1.6 |
| fak1 | ufsrat | 18 | 275 | 6.55% | 114 | 5516 | 2.07% | 3.2 |
| fkb1a | usr | 14 | 305 | 4.59% | 273 | 6105 | 4.47% | 1 |
| fkb1a | ufsrat | 19 | 305 | 6.23% | 273 | 6105 | 4.47% | 1.4 |
| fnta | usr | 66 | 2685 | 2.46% | 1692 | 53741 | 3.15% | 0.8 |
| fnta | ufsrat | 29 | 2685 | 1.08% | 1692 | 53741 | 3.15% | 0.3 |
| fpps | usr | 27 | 460 | 5.87% | 213 | 9228 | 2.31% | 2.5 |
| fpps | ufsrat | 60 | 460 | 13.04% | 213 | 9228 | 2.31% | 5.6 |
| gcr | usr | 14 | 785 | 1.78% | 563 | 15748 | 3.58% | 0.5 |
| gcr | ufsrat | 11 | 785 | 1.40% | 563 | 15748 | 3.58% | 0.4 |
| glcm | usr | 7 | 205 | 3.41% | 313 | 4150 | 7.54% | 0.5 |
| glcm | ufsrat | 9 | 205 | 4.39% | 313 | 4150 | 7.54% | 0.6 |
| gria2 | usr | 18 | 615 | 2.93% | 297 | 12358 | 2.40% | 1.2 |
| gria2 | ufsrat | 13 | 615 | 2.11% | 297 | 12358 | 2.40% | 0.9 |
| grik1 | usr | 4 | 335 | 1.19% | 152 | 6769 | 2.25% | 0.5 |
| grik1 | ufsrat | 2 | 335 | 0.60% | 152 | 6769 | 2.25% | 0.3 |
| hdac2 | usr | 1 | 530 | 0.19% | 238 | 10604 | 2.24% | 0.1 |
| hdac2 | ufsrat | 6 | 530 | 1.13% | 238 | 10604 | 2.24% | 0.5 |
| hdac8 | usr | 4 | 535 | 0.75% | 234 | 10748 | 2.18% | 0.3 |
| hdac8 | ufsrat | 5 | 535 | 0.93% | 234 | 10748 | 2.18% | 0.4 |
| hivint | usr | 3 | 345 | 0.87% | 211 | 6967 | 3.03% | 0.3 |
| hivint | ufsrat | 11 | 345 | 3.19% | 211 | 6967 | 3.03% | 1.1 |
| hivpr | usr | 85 | 1880 | 4.52% | 1395 | 37673 | 3.70% | 1.2 |
| hivpr | ufsrat | 71 | 1880 | 3.78% | 1395 | 37673 | 3.70% | 1 |
| hivrt | usr | 56 | 985 | 5.69% | 639 | 19773 | 3.23% | 1.8 |
| hivrt | ufsrat | 44 | 985 | 4.47% | 639 | 19773 | 3.23% | 1.4 |
| hmdh | usr | 25 | 455 | 5.49% | 299 | 9183 | 3.26% | 1.7 |
| hmdh | ufsrat | 10 | 455 | 2.20% | 299 | 9183 | 3.26% | 0.7 |
| hs90a | usr | 11 | 250 | 4.40% | 125 | 5067 | 2.47% | 1.8 |
| hs90a | ufsrat | 13 | 250 | 5.20% | 125 | 5067 | 2.47% | 2.1 |
| hxk4 | usr | 4 | 245 | 1.63% | 127 | 4930 | 2.58% | 0.6 |
| hxk4 | ufsrat | 22 | 245 | 8.98% | 127 | 4930 | 2.58% | 3.5 |
| igf1r | usr | 6 | 480 | 1.25% | 226 | 9633 | 2.35% | 0.5 |
| igf1r | ufsrat | 10 | 480 | 2.08% | 226 | 9633 | 2.35% | 0.9 |
| inha | usr | 0 | 115 | 0.00% | 71 | 2389 | 2.97% | 0 |
| inha | ufsrat | 8 | 115 | 6.96% | 71 | 2389 | 2.97% | 2.3 |
| ital | usr | 14 | 445 | 3.15% | 233 | 8923 | 2.61% | 1.2 |
| ital | ufsrat | 19 | 445 | 4.27% | 233 | 8923 | 2.61% | 1.6 |
| jak2 | usr | 10 | 335 | 2.99% | 153 | 6743 | 2.27% | 1.3 |
| jak2 | ufsrat | 6 | 335 | 1.79% | 153 | 6743 | 2.27% | 0.8 |
| kif11 | usr | 7 | 355 | 1.97% | 197 | 7109 | 2.77% | 0.7 |
| kif11 | ufsrat | 3 | 355 | 0.85% | 197 | 7109 | 2.77% | 0.3 |
| kit | usr | 1 | 540 | 0.19% | 252 | 10861 | 2.32% | 0.1 |
| kit | ufsrat | 3 | 540 | 0.56% | 252 | 10861 | 2.32% | 0.2 |
| kith | usr | 8 | 145 | 5.52% | 132 | 2998 | 4.40% | 1.3 |
| kith | ufsrat | 8 | 145 | 5.52% | 132 | 2998 | 4.40% | 1.3 |
| kpcb | usr | 26 | 450 | 5.78% | 248 | 9092 | 2.73% | 2.1 |
| kpcb | ufsrat | 29 | 450 | 6.44% | 248 | 9092 | 2.73% | 2.4 |
| lck | usr | 7 | 1425 | 0.49% | 683 | 28539 | 2.39% | 0.2 |
| lck | ufsrat | 35 | 1425 | 2.46% | 683 | 28539 | 2.39% | 1 |
| lkha4 | usr | 17 | 485 | 3.51% | 244 | 9721 | 2.51% | 1.4 |
| lkha4 | ufsrat | 7 | 485 | 1.44% | 244 | 9721 | 2.51% | 0.6 |
| mapk2 | usr | 4 | 320 | 1.25% | 206 | 6450 | 3.19% | 0.4 |
| mapk2 | ufsrat | 7 | 320 | 2.19% | 206 | 6450 | 3.19% | 0.7 |
| mcr | usr | 14 | 270 | 5.19% | 193 | 5433 | 3.55% | 1.5 |
| mcr | ufsrat | 21 | 270 | 7.78% | 193 | 5433 | 3.55% | 2.2 |
| met | usr | 59 | 580 | 10.17% | 244 | 11677 | 2.09% | 4.9 |
| met | ufsrat | 41 | 580 | 7.07% | 244 | 11677 | 2.09% | 3.4 |
| mk01 | usr | 4 | 235 | 1.70% | 139 | 4767 | 2.92% | 0.6 |
| mk01 | ufsrat | 7 | 235 | 2.98% | 139 | 4767 | 2.92% | 1 |
| mk10 | usr | 3 | 345 | 0.87% | 186 | 6900 | 2.70% | 0.3 |
| mk10 | ufsrat | 2 | 345 | 0.58% | 186 | 6900 | 2.70% | 0.2 |
| mk14 | usr | 55 | 1865 | 2.95% | 915 | 37347 | 2.45% | 1.2 |
| mk14 | ufsrat | 19 | 1865 | 1.02% | 915 | 37347 | 2.45% | 0.4 |
| mmp13 | usr | 49 | 1950 | 2.51% | 1038 | 39046 | 2.66% | 0.9 |
| mmp13 | ufsrat | 105 | 1950 | 5.38% | 1038 | 39046 | 2.66% | 2 |
| mp2k1 | usr | 21 | 420 | 5.00% | 242 | 8483 | 2.85% | 1.8 |
| mp2k1 | ufsrat | 21 | 420 | 5.00% | 242 | 8483 | 2.85% | 1.8 |
| nos1 | usr | 2 | 415 | 0.48% | 234 | 8307 | 2.82% | 0.2 |
| nos1 | ufsrat | 3 | 415 | 0.72% | 234 | 8307 | 2.82% | 0.3 |
| nram | usr | 43 | 320 | 13.44% | 222 | 6449 | 3.44% | 3.9 |
| nram | ufsrat | 57 | 320 | 17.81% | 222 | 6449 | 3.44% | 5.2 |
| pa2ga | usr | 4 | 265 | 1.51% | 127 | 5343 | 2.38% | 0.6 |
| pa2ga | ufsrat | 38 | 265 | 14.34% | 127 | 5343 | 2.38% | 6 |
| parp1 | usr | 45 | 1555 | 2.89% | 742 | 31171 | 2.38% | 1.2 |
| parp1 | ufsrat | 44 | 1555 | 2.83% | 742 | 31171 | 2.38% | 1.2 |
| pde5a | usr | 47 | 1425 | 3.30% | 706 | 28532 | 2.47% | 1.3 |
| pde5a | ufsrat | 43 | 1425 | 3.02% | 706 | 28532 | 2.47% | 1.2 |
| pgh1 | usr | 21 | 555 | 3.78% | 251 | 11193 | 2.24% | 1.7 |
| pgh1 | ufsrat | 20 | 555 | 3.60% | 251 | 11193 | 2.24% | 1.6 |
| pgh2 | usr | 85 | 1195 | 7.11% | 531 | 23936 | 2.22% | 3.2 |
| pgh2 | ufsrat | 67 | 1195 | 5.61% | 531 | 23936 | 2.22% | 2.5 |
| plk1 | usr | 10 | 350 | 2.86% | 155 | 7034 | 2.20% | 1.3 |
| plk1 | ufsrat | 8 | 350 | 2.29% | 155 | 7034 | 2.20% | 1 |
| pnph | usr | 22 | 360 | 6.11% | 233 | 7249 | 3.21% | 1.9 |
| pnph | ufsrat | 19 | 360 | 5.28% | 233 | 7249 | 3.21% | 1.6 |
| ppara | usr | 85 | 1015 | 8.37% | 544 | 20375 | 2.67% | 3.1 |
| ppara | ufsrat | 31 | 1015 | 3.05% | 544 | 20375 | 2.67% | 1.1 |
| ppard | usr | 3 | 675 | 0.44% | 288 | 13520 | 2.13% | 0.2 |
| ppard | ufsrat | 9 | 675 | 1.33% | 288 | 13520 | 2.13% | 0.6 |
| pparg | usr | 24 | 1325 | 1.81% | 723 | 26590 | 2.72% | 0.7 |
| pparg | ufsrat | 30 | 1325 | 2.26% | 723 | 26590 | 2.72% | 0.8 |
| prgr | usr | 58 | 810 | 7.16% | 444 | 16258 | 2.73% | 2.6 |
| prgr | ufsrat | 56 | 810 | 6.91% | 444 | 16258 | 2.73% | 2.5 |
| ptn1 | usr | 3 | 380 | 0.79% | 225 | 7658 | 2.94% | 0.3 |
| ptn1 | ufsrat | 3 | 380 | 0.79% | 225 | 7658 | 2.94% | 0.3 |
| pur2 | usr | 0 | 145 | 0.00% | 201 | 2926 | 6.87% | 0 |
| pur2 | ufsrat | 1 | 145 | 0.69% | 201 | 2926 | 6.87% | 0.1 |
| pygm | usr | 11 | 205 | 5.37% | 114 | 4159 | 2.74% | 2 |
| pygm | ufsrat | 19 | 205 | 9.27% | 114 | 4159 | 2.74% | 3.4 |
| pyrd | usr | 29 | 335 | 8.66% | 134 | 6782 | 1.98% | 4.4 |
| pyrd | ufsrat | 34 | 335 | 10.15% | 134 | 6782 | 1.98% | 5.1 |
| reni | usr | 6 | 365 | 1.64% | 387 | 7371 | 5.25% | 0.3 |
| reni | ufsrat | 10 | 365 | 2.74% | 387 | 7371 | 5.25% | 0.5 |
| rock1 | usr | 10 | 325 | 3.08% | 203 | 6580 | 3.09% | 1 |
| rock1 | ufsrat | 11 | 325 | 3.38% | 203 | 6580 | 3.09% | 1.1 |
| rxra | usr | 2 | 390 | 0.51% | 162 | 7869 | 2.06% | 0.2 |
| rxra | ufsrat | 18 | 390 | 4.62% | 162 | 7869 | 2.06% | 2.2 |
| sahh | usr | 20 | 180 | 11.11% | 190 | 3673 | 5.17% | 2.1 |
| sahh | ufsrat | 16 | 180 | 8.89% | 190 | 3673 | 5.17% | 1.7 |
| src | usr | 22 | 1785 | 1.23% | 831 | 35790 | 2.32% | 0.5 |
| src | ufsrat | 85 | 1785 | 4.76% | 831 | 35790 | 2.32% | 2.1 |
| tgfr1 | usr | 16 | 445 | 3.60% | 281 | 8958 | 3.14% | 1.1 |
| tgfr1 | ufsrat | 51 | 445 | 11.46% | 281 | 8958 | 3.14% | 3.6 |
| thb | usr | 4 | 390 | 1.03% | 168 | 7821 | 2.15% | 0.5 |
| thb | ufsrat | 41 | 390 | 10.51% | 168 | 7821 | 2.15% | 4.9 |
| thrb | usr | 18 | 1405 | 1.28% | 861 | 28182 | 3.06% | 0.4 |
| thrb | ufsrat | 17 | 1405 | 1.21% | 861 | 28182 | 3.06% | 0.4 |
| try1 | usr | 58 | 1345 | 4.31% | 758 | 26977 | 2.81% | 1.5 |
| try1 | ufsrat | 18 | 1345 | 1.34% | 758 | 26977 | 2.81% | 0.5 |
| tryb1 | usr | 6 | 390 | 1.54% | 171 | 7884 | 2.17% | 0.7 |
| tryb1 | ufsrat | 0 | 390 | 0.00% | 171 | 7884 | 2.17% | 0 |
| tysy | usr | 27 | 355 | 7.61% | 311 | 7194 | 4.32% | 1.8 |
| tysy | ufsrat | 23 | 355 | 6.48% | 311 | 7194 | 4.32% | 1.5 |
| urok | usr | 7 | 510 | 1.37% | 306 | 10239 | 2.99% | 0.5 |
| urok | ufsrat | 15 | 510 | 2.94% | 306 | 10239 | 2.99% | 1 |
| vgfr2 | usr | 36 | 1295 | 2.78% | 620 | 25900 | 2.39% | 1.2 |
| vgfr2 | ufsrat | 4 | 1295 | 0.31% | 620 | 25900 | 2.39% | 0.1 |
| wee1 | usr | 0 | 315 | 0.00% | 137 | 6371 | 2.15% | 0 |
| wee1 | ufsrat | 3 | 315 | 0.95% | 137 | 6371 | 2.15% | 0.4 |
| xiap | usr | 13 | 265 | 4.91% | 129 | 5342 | 2.41% | 2 |
| xiap | ufsrat | 12 | 265 | 4.53% | 129 | 5342 | 2.41% | 1.9 |
