# Supplementary material for: UFSRAT: Ultra-Fast Shape Recognition with Atom Types –The Discovery of Novel Bioactive Small Molecular Scaffolds for FKBP12 and 11βHSD1
Source: PLoS One. 2015 Feb 6;10(2):e0116570. doi: 10.1371/journal.pone.0116570 (PMC4319890; doi:10.1371/journal.pone.0116570)
Supplement: S6 Table — (DOCX) [file pone.0116570.s010.docx]

Table S6 - DUD-E profiling of ECFP4 at the 0.5, 1, 2 and 5% levels

|  |  |  | Hits | | | |  | Success rate | | | |  | Enrichment | | | |
| --- | --- | --- | --- | --- | --- | --- | --- | --- | --- | --- | --- | --- | --- | --- | --- | --- |
| Target | Library actives | Library total | 0.5% level | 1% level | 2% level | 5% level | Possible hits | 0.5% level | 1% level | 2% level | 5% level | Library actives proportion | 0.5% level | 1% level | 2% level | 5% level |
| aa2ar | 844 | 32908 | 156 | 230 | 264 | 308 | 164 | 0.95 | 0.70 | 0.40 | 0.19 | 2.56% | 37.0 | 27.3 | 15.7 | 7.3 |
| abl1 | 295 | 11180 | 23 | 34 | 48 | 71 | 55 | 0.41 | 0.30 | 0.21 | 0.13 | 2.64% | 15.6 | 11.5 | 8.1 | 4.8 |
| ace | 808 | 17952 | 75 | 101 | 124 | 152 | 89 | 0.84 | 0.56 | 0.35 | 0.17 | 4.50% | 18.6 | 12.5 | 7.7 | 3.8 |
| aces | 664 | 27037 | 18 | 25 | 44 | 74 | 135 | 0.13 | 0.09 | 0.08 | 0.05 | 2.46% | 5.4 | 3.8 | 3.3 | 2.2 |
| ada | 262 | 5734 | 27 | 37 | 40 | 46 | 28 | 0.94 | 0.65 | 0.35 | 0.16 | 4.57% | 20.6 | 14.1 | 7.6 | 3.5 |
| ada17 | 959 | 37606 | 111 | 141 | 170 | 219 | 188 | 0.59 | 0.37 | 0.23 | 0.12 | 2.55% | 23.2 | 14.7 | 8.9 | 4.6 |
| adrb1 | 458 | 16416 | 21 | 29 | 35 | 57 | 82 | 0.26 | 0.18 | 0.11 | 0.07 | 2.79% | 9.2 | 6.3 | 3.8 | 2.5 |
| adrb2 | 447 | 15702 | 6 | 9 | 14 | 26 | 78 | 0.08 | 0.06 | 0.04 | 0.03 | 2.85% | 2.7 | 2.0 | 1.6 | 1.2 |
| akt1 | 423 | 16999 | 19 | 30 | 42 | 77 | 84 | 0.22 | 0.18 | 0.12 | 0.09 | 2.49% | 9.0 | 7.1 | 5.0 | 3.6 |
| akt2 | 190 | 7142 | 12 | 12 | 12 | 12 | 35 | 0.34 | 0.17 | 0.08 | 0.03 | 2.66% | 12.6 | 6.3 | 3.2 | 1.3 |
| aldr | 220 | 9356 | 42 | 61 | 71 | 79 | 46 | 0.90 | 0.65 | 0.38 | 0.17 | 2.35% | 38.2 | 27.7 | 16.1 | 7.2 |
| ampc | 62 | 2964 | 0 | 0 | 0 | 0 | 14 | 0.00 | 0.00 | 0.00 | 0.00 | 2.09% | 0.0 | 0.0 | 0.0 | 0.0 |
| andr | 523 | 15026 | 38 | 58 | 60 | 66 | 75 | 0.51 | 0.39 | 0.20 | 0.09 | 3.48% | 14.5 | 11.1 | 5.7 | 2.5 |
| aofb | 168 | 7099 | 4 | 5 | 7 | 7 | 35 | 0.11 | 0.07 | 0.05 | 0.02 | 2.37% | 4.8 | 3.0 | 2.1 | 0.8 |
| bace1 | 485 | 18706 | 1 | 2 | 4 | 8 | 93 | 0.01 | 0.01 | 0.01 | 0.01 | 2.59% | 0.4 | 0.4 | 0.4 | 0.3 |
| braf | 251 | 10349 | 11 | 15 | 22 | 33 | 51 | 0.21 | 0.14 | 0.11 | 0.06 | 2.43% | 8.7 | 6.0 | 4.4 | 2.6 |
| cah2 | 835 | 32545 | 3 | 7 | 14 | 78 | 162 | 0.02 | 0.02 | 0.02 | 0.05 | 2.57% | 0.7 | 0.8 | 0.8 | 1.9 |
| casp3 | 350 | 11172 | 29 | 41 | 58 | 89 | 55 | 0.52 | 0.37 | 0.26 | 0.16 | 3.13% | 16.6 | 11.7 | 8.3 | 5.1 |
| cdk2 | 798 | 29126 | 8 | 16 | 28 | 57 | 145 | 0.05 | 0.05 | 0.05 | 0.04 | 2.74% | 2.0 | 2.0 | 1.8 | 1.4 |
| comt | 86 | 4012 | 18 | 31 | 36 | 37 | 20 | 0.90 | 0.77 | 0.45 | 0.18 | 2.14% | 41.9 | 36.1 | 21.0 | 8.6 |
| cp2c9 | 183 | 7757 | 2 | 3 | 5 | 10 | 38 | 0.05 | 0.04 | 0.03 | 0.03 | 2.36% | 2.2 | 1.6 | 1.4 | 1.1 |
| cp3a4 | 363 | 12303 | 6 | 6 | 8 | 12 | 61 | 0.10 | 0.05 | 0.03 | 0.02 | 2.95% | 3.3 | 1.7 | 1.1 | 0.7 |
| csf1r | 286 | 12720 | 23 | 25 | 30 | 34 | 63 | 0.36 | 0.20 | 0.12 | 0.05 | 2.25% | 16.1 | 8.7 | 5.2 | 2.4 |
| cxcr4 | 122 | 3536 | 15 | 15 | 15 | 15 | 17 | 0.85 | 0.42 | 0.21 | 0.08 | 3.45% | 24.6 | 12.3 | 6.1 | 2.5 |
| def | 161 | 5899 | 21 | 32 | 48 | 64 | 29 | 0.71 | 0.54 | 0.41 | 0.22 | 2.73% | 26.1 | 19.9 | 14.9 | 7.9 |
| dhi1 | 519 | 20142 | 29 | 51 | 79 | 128 | 100 | 0.29 | 0.25 | 0.20 | 0.13 | 2.58% | 11.2 | 9.8 | 7.6 | 4.9 |
| dpp4 | 1079 | 42452 | 95 | 109 | 129 | 171 | 212 | 0.45 | 0.26 | 0.15 | 0.08 | 2.54% | 17.6 | 10.1 | 6.0 | 3.2 |
| drd3 | 877 | 35065 | 2 | 2 | 4 | 8 | 175 | 0.01 | 0.01 | 0.01 | 0.00 | 2.50% | 0.5 | 0.2 | 0.2 | 0.2 |
| dyr | 566 | 17950 | 86 | 120 | 140 | 179 | 89 | 0.96 | 0.67 | 0.39 | 0.20 | 3.15% | 30.4 | 21.2 | 12.4 | 6.3 |
| egfr | 832 | 36274 | 163 | 253 | 302 | 365 | 181 | 0.90 | 0.70 | 0.42 | 0.20 | 2.29% | 39.2 | 30.5 | 18.2 | 8.8 |
| esr1 | 627 | 21445 | 102 | 153 | 191 | 226 | 107 | 0.95 | 0.71 | 0.45 | 0.21 | 2.92% | 32.6 | 24.4 | 15.3 | 7.2 |
| esr2 | 595 | 20908 | 40 | 59 | 92 | 147 | 104 | 0.38 | 0.28 | 0.22 | 0.14 | 2.85% | 13.4 | 9.9 | 7.7 | 4.9 |
| fa10 | 792 | 21209 | 26 | 39 | 49 | 66 | 106 | 0.25 | 0.18 | 0.12 | 0.06 | 3.73% | 6.6 | 4.9 | 3.1 | 1.7 |
| fa7 | 185 | 6487 | 21 | 29 | 39 | 61 | 32 | 0.65 | 0.45 | 0.30 | 0.19 | 2.85% | 22.7 | 15.7 | 10.5 | 6.6 |
| fabp4 | 57 | 2912 | 13 | 15 | 15 | 19 | 14 | 0.89 | 0.52 | 0.26 | 0.13 | 1.96% | 45.6 | 26.3 | 13.1 | 6.7 |
| fak1 | 114 | 5516 | 26 | 33 | 47 | 79 | 27 | 0.94 | 0.60 | 0.43 | 0.29 | 2.07% | 45.5 | 28.9 | 20.6 | 13.8 |
| fkb1a | 273 | 6105 | 12 | 16 | 21 | 27 | 30 | 0.07 | 0.26 | 0.17 | 0.09 | 4.47% | 1.5 | 5.9 | 3.8 | 2.0 |
| fnta | 1692 | 53741 | 32 | 44 | 58 | 97 | 268 | 0.01 | 0.08 | 0.05 | 0.04 | 3.15% | 0.4 | 2.6 | 1.7 | 1.1 |
| fpps | 213 | 9228 | 44 | 80 | 83 | 84 | 46 | 0.48 | 0.87 | 0.45 | 0.18 | 2.31% | 20.7 | 37.5 | 19.5 | 7.9 |
| gcr | 563 | 15748 | 40 | 51 | 71 | 101 | 78 | 0.03 | 0.32 | 0.23 | 0.13 | 3.58% | 0.7 | 9.0 | 6.3 | 3.6 |
| glcm | 313 | 4150 | 2 | 7 | 12 | 20 | 20 | 0.25 | 0.17 | 0.14 | 0.10 | 7.54% | 3.3 | 2.2 | 1.9 | 1.3 |
| gria2 | 297 | 12358 | 10 | 15 | 16 | 20 | 61 | 0.03 | 0.12 | 0.06 | 0.03 | 2.40% | 1.4 | 5.1 | 2.7 | 1.3 |
| grik1 | 152 | 6769 | 2 | 3 | 4 | 5 | 33 | 0.00 | 0.04 | 0.03 | 0.01 | 2.25% | 0.0 | 2.0 | 1.3 | 0.7 |
| hdac2 | 238 | 10604 | 31 | 34 | 43 | 57 | 53 | 0.06 | 0.32 | 0.20 | 0.11 | 2.24% | 2.5 | 14.3 | 9.1 | 4.8 |
| hdac8 | 234 | 10748 | 35 | 47 | 59 | 89 | 53 | 0.08 | 0.44 | 0.27 | 0.17 | 2.18% | 3.5 | 20.1 | 12.6 | 7.6 |
| hivint | 211 | 6967 | 0 | 1 | 1 | 11 | 34 | 0.03 | 0.01 | 0.01 | 0.03 | 3.03% | 1.0 | 0.5 | 0.2 | 1.0 |
| hivpr | 1395 | 37673 | 99 | 142 | 183 | 266 | 188 | 0.04 | 0.38 | 0.24 | 0.14 | 3.70% | 1.0 | 10.2 | 6.6 | 3.8 |
| hivrt | 639 | 19773 | 16 | 22 | 25 | 38 | 98 | 0.10 | 0.11 | 0.06 | 0.04 | 3.23% | 3.2 | 3.4 | 2.0 | 1.2 |
| hmdh | 299 | 9183 | 44 | 89 | 122 | 126 | 45 | 0.11 | 0.97 | 0.66 | 0.27 | 3.26% | 3.4 | 29.7 | 20.4 | 8.4 |
| hs90a | 125 | 5067 | 15 | 17 | 28 | 49 | 25 | 0.00 | 0.34 | 0.28 | 0.19 | 2.47% | 0.0 | 13.6 | 11.2 | 7.8 |
| hxk4 | 127 | 4930 | 20 | 29 | 43 | 58 | 24 | 0.29 | 0.59 | 0.44 | 0.24 | 2.58% | 11.3 | 22.8 | 16.9 | 9.1 |
| igf1r | 226 | 9633 | 22 | 24 | 30 | 58 | 48 | 0.06 | 0.25 | 0.16 | 0.12 | 2.35% | 2.7 | 10.6 | 6.6 | 5.1 |
| inha | 71 | 2389 | 7 | 7 | 8 | 9 | 11 | 0.36 | 0.29 | 0.17 | 0.08 | 2.97% | 12.2 | 9.9 | 5.6 | 2.5 |
| ital | 233 | 8923 | 20 | 21 | 26 | 31 | 44 | 0.14 | 0.24 | 0.15 | 0.07 | 2.61% | 5.2 | 9.0 | 5.6 | 2.7 |
| jak2 | 153 | 6743 | 14 | 18 | 22 | 35 | 33 | 0.12 | 0.27 | 0.16 | 0.10 | 2.27% | 5.3 | 11.8 | 7.2 | 4.6 |
| kif11 | 197 | 7109 | 21 | 28 | 34 | 48 | 35 | 0.00 | 0.39 | 0.24 | 0.14 | 2.77% | 0.0 | 14.2 | 8.6 | 4.9 |
| kit | 252 | 10861 | 2 | 2 | 2 | 4 | 54 | 0.00 | 0.02 | 0.01 | 0.01 | 2.32% | 0.0 | 0.8 | 0.4 | 0.3 |
| kith | 132 | 2998 | 14 | 29 | 40 | 42 | 14 | 0.36 | 0.97 | 0.67 | 0.28 | 4.40% | 8.1 | 22.0 | 15.2 | 6.4 |
| kpcb | 248 | 9092 | 44 | 71 | 83 | 88 | 45 | 0.13 | 0.78 | 0.46 | 0.19 | 2.73% | 4.9 | 28.6 | 16.7 | 7.1 |
| lck | 683 | 28539 | 20 | 23 | 44 | 79 | 142 | 0.06 | 0.08 | 0.08 | 0.06 | 2.39% | 2.7 | 3.4 | 3.2 | 2.3 |
| lkha4 | 244 | 9721 | 23 | 35 | 42 | 63 | 48 | 0.02 | 0.36 | 0.22 | 0.13 | 2.51% | 0.8 | 14.3 | 8.6 | 5.2 |
| mapk2 | 206 | 6450 | 14 | 15 | 17 | 17 | 32 | 0.00 | 0.23 | 0.13 | 0.05 | 3.19% | 0.0 | 7.3 | 4.1 | 1.7 |
| mcr | 193 | 5433 | 11 | 12 | 12 | 14 | 27 | 0.37 | 0.22 | 0.11 | 0.05 | 3.55% | 10.4 | 6.2 | 3.1 | 1.5 |
| met | 244 | 11677 | 42 | 60 | 69 | 81 | 58 | 0.22 | 0.51 | 0.30 | 0.14 | 2.09% | 10.7 | 24.6 | 14.1 | 6.6 |
| mk01 | 139 | 4767 | 23 | 29 | 41 | 56 | 23 | 0.04 | 0.61 | 0.43 | 0.23 | 2.92% | 1.5 | 20.8 | 14.7 | 8.0 |
| mk10 | 186 | 6900 | 4 | 7 | 8 | 13 | 34 | 0.00 | 0.10 | 0.06 | 0.04 | 2.70% | 0.0 | 3.8 | 2.1 | 1.4 |
| mk14 | 915 | 37347 | 10 | 13 | 19 | 45 | 186 | 0.01 | 0.03 | 0.03 | 0.02 | 2.45% | 0.4 | 1.4 | 1.0 | 1.0 |
| mmp13 | 1038 | 39046 | 144 | 196 | 248 | 325 | 195 | 0.05 | 0.50 | 0.32 | 0.17 | 2.66% | 1.9 | 18.9 | 11.9 | 6.3 |
| mp2k1 | 242 | 8483 | 24 | 27 | 39 | 56 | 42 | 0.26 | 0.32 | 0.23 | 0.13 | 2.85% | 9.2 | 11.2 | 8.1 | 4.6 |
| nos1 | 234 | 8307 | 0 | 0 | 0 | 0 | 41 | 0.00 | 0.00 | 0.00 | 0.00 | 2.82% | 0.0 | 0.0 | 0.0 | 0.0 |
| nram | 222 | 6449 | 10 | 12 | 20 | 28 | 32 | 0.53 | 0.19 | 0.16 | 0.09 | 3.44% | 15.4 | 5.4 | 4.5 | 2.5 |
| pa2ga | 127 | 5343 | 4 | 8 | 11 | 19 | 26 | 0.08 | 0.15 | 0.10 | 0.07 | 2.38% | 3.2 | 6.3 | 4.3 | 3.0 |
| parp1 | 742 | 31171 | 51 | 62 | 88 | 135 | 155 | 0.03 | 0.20 | 0.14 | 0.09 | 2.38% | 1.4 | 8.4 | 5.9 | 3.6 |
| pde5a | 706 | 28532 | 49 | 52 | 55 | 66 | 142 | 0.07 | 0.18 | 0.10 | 0.05 | 2.47% | 2.9 | 7.4 | 3.9 | 1.9 |
| pgh1 | 251 | 11193 | 4 | 9 | 13 | 21 | 55 | 0.02 | 0.08 | 0.06 | 0.04 | 2.24% | 0.8 | 3.6 | 2.6 | 1.7 |
| pgh2 | 531 | 23936 | 88 | 114 | 144 | 190 | 119 | 0.34 | 0.48 | 0.30 | 0.16 | 2.22% | 15.5 | 21.5 | 13.5 | 7.2 |
| plk1 | 155 | 7034 | 1 | 1 | 1 | 1 | 35 | 0.00 | 0.01 | 0.01 | 0.00 | 2.20% | 0.0 | 0.6 | 0.3 | 0.1 |
| pnph | 233 | 7249 | 32 | 38 | 42 | 46 | 36 | 0.22 | 0.52 | 0.29 | 0.13 | 3.21% | 6.9 | 16.3 | 9.0 | 4.0 |
| ppara | 544 | 20375 | 82 | 148 | 197 | 262 | 101 | 0.08 | 0.73 | 0.48 | 0.26 | 2.67% | 3.0 | 27.2 | 18.1 | 9.6 |
| ppard | 288 | 13520 | 23 | 33 | 51 | 91 | 67 | 0.00 | 0.24 | 0.19 | 0.13 | 2.13% | 0.0 | 11.5 | 8.9 | 6.3 |
| pparg | 723 | 26590 | 82 | 120 | 174 | 245 | 132 | 0.03 | 0.45 | 0.33 | 0.18 | 2.72% | 1.1 | 16.6 | 12.0 | 6.8 |
| prgr | 444 | 16258 | 17 | 19 | 24 | 34 | 81 | 0.21 | 0.12 | 0.07 | 0.04 | 2.73% | 7.7 | 4.3 | 2.7 | 1.5 |
| ptn1 | 225 | 7658 | 20 | 31 | 32 | 38 | 38 | 0.03 | 0.40 | 0.21 | 0.10 | 2.94% | 0.9 | 13.8 | 7.1 | 3.4 |
| pur2 | 201 | 2926 | 13 | 27 | 49 | 49 | 14 | 0.00 | 0.92 | 0.84 | 0.33 | 6.87% | 0.0 | 13.4 | 12.2 | 4.9 |
| pygm | 114 | 4159 | 0 | 0 | 0 | 6 | 20 | 0.40 | 0.00 | 0.00 | 0.03 | 2.74% | 14.6 | 0.0 | 0.0 | 1.1 |
| pyrd | 134 | 6782 | 32 | 54 | 55 | 63 | 33 | 0.67 | 0.80 | 0.41 | 0.19 | 1.98% | 33.7 | 40.2 | 20.5 | 9.4 |
| reni | 387 | 7371 | 4 | 4 | 6 | 8 | 36 | 0.06 | 0.05 | 0.04 | 0.02 | 5.25% | 1.1 | 1.0 | 0.8 | 0.4 |
| rock1 | 203 | 6580 | 0 | 0 | 0 | 0 | 32 | 0.00 | 0.00 | 0.00 | 0.00 | 3.09% | 0.0 | 0.0 | 0.0 | 0.0 |
| rxra | 162 | 7869 | 5 | 8 | 18 | 34 | 39 | 0.08 | 0.10 | 0.11 | 0.09 | 2.06% | 3.7 | 4.9 | 5.6 | 4.2 |
| sahh | 190 | 3673 | 17 | 35 | 62 | 62 | 18 | 0.28 | 0.95 | 0.84 | 0.34 | 5.17% | 5.4 | 18.4 | 16.3 | 6.5 |
| src | 831 | 35790 | 45 | 55 | 70 | 99 | 178 | 0.07 | 0.15 | 0.10 | 0.06 | 2.32% | 2.9 | 6.6 | 4.2 | 2.4 |
| tgfr1 | 281 | 8958 | 40 | 54 | 67 | 109 | 44 | 0.30 | 0.60 | 0.37 | 0.24 | 3.14% | 9.4 | 19.2 | 11.9 | 7.8 |
| thb | 168 | 7821 | 20 | 23 | 25 | 27 | 39 | 0.23 | 0.29 | 0.16 | 0.07 | 2.15% | 10.7 | 13.7 | 7.4 | 3.2 |
| thrb | 861 | 28182 | 0 | 0 | 0 | 0 | 140 | 0.01 | 0.00 | 0.00 | 0.00 | 3.06% | 0.2 | 0.0 | 0.0 | 0.0 |
| try1 | 758 | 26977 | 0 | 0 | 0 | 0 | 134 | 0.01 | 0.00 | 0.00 | 0.00 | 2.81% | 0.3 | 0.0 | 0.0 | 0.0 |
| tryb1 | 171 | 7884 | 1 | 2 | 4 | 23 | 39 | 0.00 | 0.03 | 0.03 | 0.06 | 2.17% | 0.0 | 1.2 | 1.2 | 2.7 |
| tysy | 311 | 7194 | 26 | 38 | 45 | 64 | 35 | 0.29 | 0.53 | 0.31 | 0.18 | 4.32% | 6.6 | 12.2 | 7.2 | 4.1 |
| urok | 306 | 10239 | 0 | 0 | 0 | 0 | 51 | 0.02 | 0.00 | 0.00 | 0.00 | 2.99% | 0.7 | 0.0 | 0.0 | 0.0 |
| vgfr2 | 620 | 25900 | 67 | 97 | 124 | 164 | 129 | 0.00 | 0.37 | 0.24 | 0.13 | 2.39% | 0.0 | 15.7 | 10.0 | 5.3 |
| wee1 | 137 | 6371 | 31 | 62 | 89 | 92 | 31 | 0.00 | 0.97 | 0.70 | 0.29 | 2.15% | 0.0 | 45.3 | 32.5 | 13.4 |
| xiap | 129 | 5342 | 25 | 50 | 79 | 88 | 26 | 0.00 | 0.94 | 0.74 | 0.33 | 2.41% | 0.0 | 38.8 | 30.7 | 13.7 |
